# X-ray microtomography–based atlas of mouse cranial development

**DOI:** 10.1093/gigascience/giab012

**Published:** 2021-03-02

**Authors:** Jan Matula, Marketa Tesarova, Tomas Zikmund, Marketa Kaucka, Igor Adameyko, Jozef Kaiser

**Affiliations:** Central European Institute of Technology, Brno University of Technology, Purkyňova 123, Brno, 61200, Czech Republic; Central European Institute of Technology, Brno University of Technology, Purkyňova 123, Brno, 61200, Czech Republic; Central European Institute of Technology, Brno University of Technology, Purkyňova 123, Brno, 61200, Czech Republic; Max Planck Institute for Evolutionary Biology, August-Thienemann-Str. 2, Plön, 24306, Germany; Medical University of Vienna, Spitalgasse 23, Vienna, 1090, Austria; Medical University of Vienna, Spitalgasse 23, Vienna, 1090, Austria; Central European Institute of Technology, Brno University of Technology, Purkyňova 123, Brno, 61200, Czech Republic

**Keywords:** X-ray, computed tomography, microtomography, mouse embryo head, tissue contrast, 3D modelling, nasal capsule

## Abstract

**Background:**

X-ray microtomography (μCT) has become an invaluable tool for non-destructive analysis of biological samples in the field of developmental biology. Mouse embryos are a typical model for investigation of human developmental diseases. By obtaining 3D high-resolution scans of the mouse embryo heads, we gain valuable morphological information about the structures prominent in the development of future face, brain, and sensory organs. The development of facial skeleton tracked in these μCT data provides a valuable background for further studies of congenital craniofacial diseases and normal development.

**Findings:**

In this work, reusable tomographic data from 7 full 3D scans of mouse embryo heads are presented and made publicly available. The ages of these embryos range from E12.5 to E18.5. The samples were stained by phosphotungstic acid prior to scanning, which greatly enhanced the contrast of various tissues in the reconstructed images and enabled precise segmentation. The images were obtained on a laboratory-based μCT system. Furthermore, we provide manually segmented masks of mesenchymal condensations (for E12.5 and E13.5) and cartilage present in the nasal capsule of the scanned embryos.

**Conclusion:**

We present a comprehensive dataset of X-ray 3D computed tomography images of the developing mouse head with high-quality manual segmentation masks of cartilaginous nasal capsules. The provided μCT images can be used for studying any other major structure within the developing mouse heads. The high quality of the manually segmented models of nasal capsules may be instrumental to understanding the complex process of the development of the face in a mouse model.

## Background

The vertebrate head is considered one of the most complex parts of the body. The head is formed during embryonic development through a process known as morphogenesis, which involves hundreds of genes and non-coding regulatory sequences [1,2]. This intricate body compartment hosts numerous cell and tissue types forming, e.g., muscles, ligaments, nerves and central nervous system, sensory organs, hair follicles, and teeth, which are all integrated in the complexly shaped skull. There is a remarkable interspecies but in some cases (such as humans) also intraspecies variability of the craniofacial shapes [3]. Reportedly, the shape of the face (or the whole head) depends on the geometry of the skeleton, which provides protection to sensitive nervous tissues and serves as a scaffold for muscle attachment [1]. The skeleton of the head is formed by 2 types of stiff tissue—bone and cartilage. Although the majority of the head skeleton in mammals is formed by bones postnatally, the embryonic development of the skull relies on the cartilage. Chondrocranium is induced as 14 independent pieces that grow, acquire specific shape, and fuse later to form the skull [1]. Interestingly, the development of cartilage and bone corresponds to the progress of development of the central and peripheral nervous system and sensory organs [2]. Therefore, the exact developmental link between the emergence of nervous structures and the appearance of cartilage and bone is one of the fundamental questions in developmental biology. At the same time, understanding both the molecular basis and cellular dynamics driving the formation and shaping of the mammalian head is of utmost interest in the fields of clinical genetics and regenerative medicine, which deal with a broad spectrum of human congenital craniofacial disorders.

In our previous work, we aimed to explore the exact sequence of formation and shaping of the developing mammalian face and we used a mouse model for our investigation [1,2]. The morphological properties of the observed structures are complex, and to fully understand their shaping, advanced imaging techniques are required. X-ray microtomography (μCT) technique is one of the oldest imaging techniques, but in recent years it has shown its strengths in the field of developmental biology [4]. The principle of μCT lies in acquiring 2D projections of the scanned sample at regular angle increments. A 3D view of the scene is then created by the process of tomographic reconstruction. This way we gain 3D spatial information that would be otherwise unobtainable without destroying the sample. The superior resolution of modern laboratory-based μCT machines provides a way to visualize and analyse biological structures on the level of microns and, more importantly, in the 3D spatial context. We combined genetic tracing, gene knockout strategies, mathematical modelling, and μCT to reconstruct craniofacial development in detail. As a result, we generated a set of μCT scans from wild-type mouse strains, ranging from E12.5 (where the first induction of early cartilage, represented by condensation of the mesenchyme, can be observed) to E18.5 with fully formed chondrocranium.

**Figure 1:**
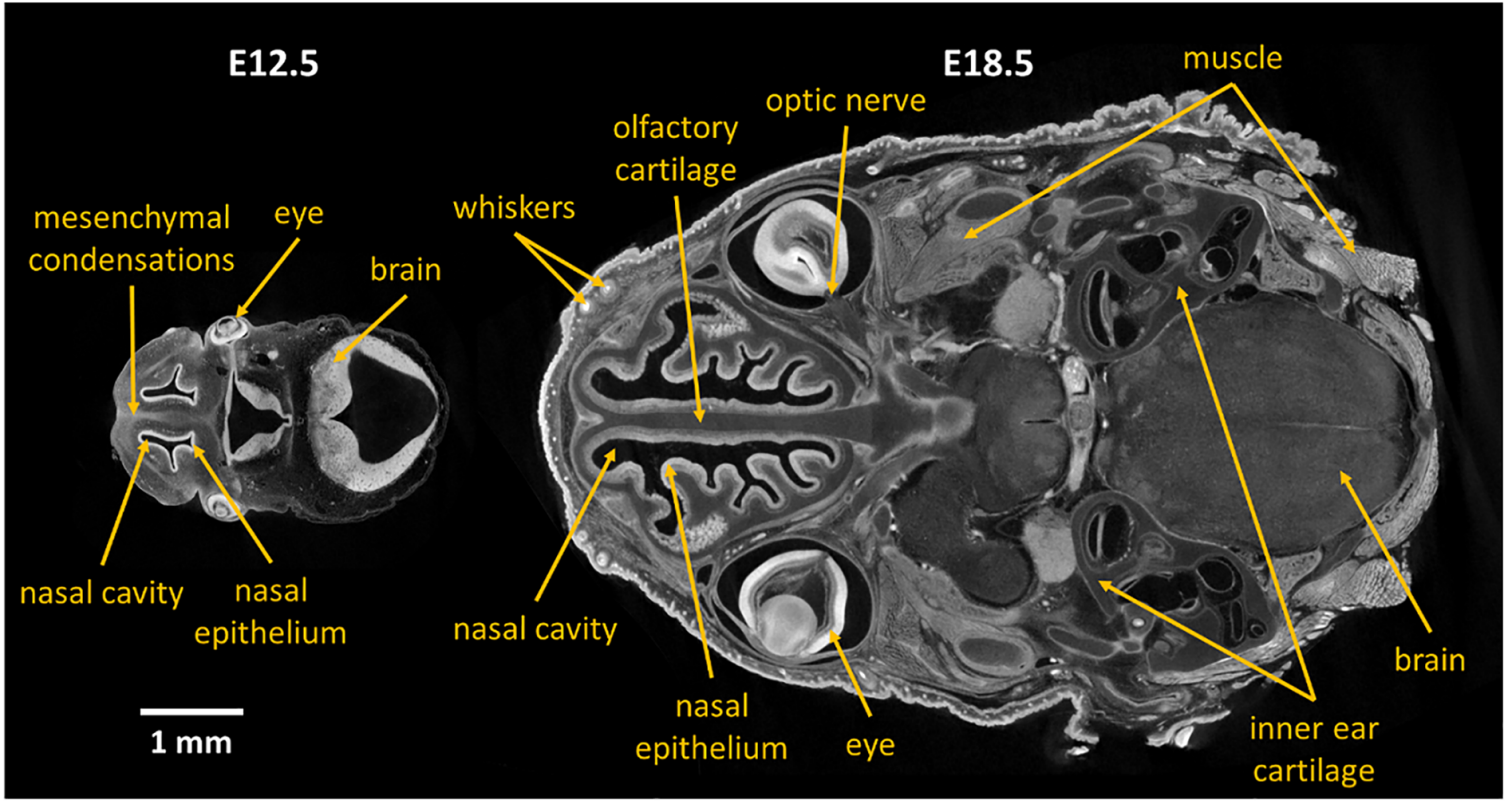
Examples of tomographic slices of mouse embryos 12.5 days old (E12.5) and 18.5 days old (E18.5). μCT scanning of samples stained with PTA provides image data with excellent contrast, where even fine details are visible. Arrows indicate areas of the image that might be interesting for potential users of the provided dataset.

**Figure 2:**
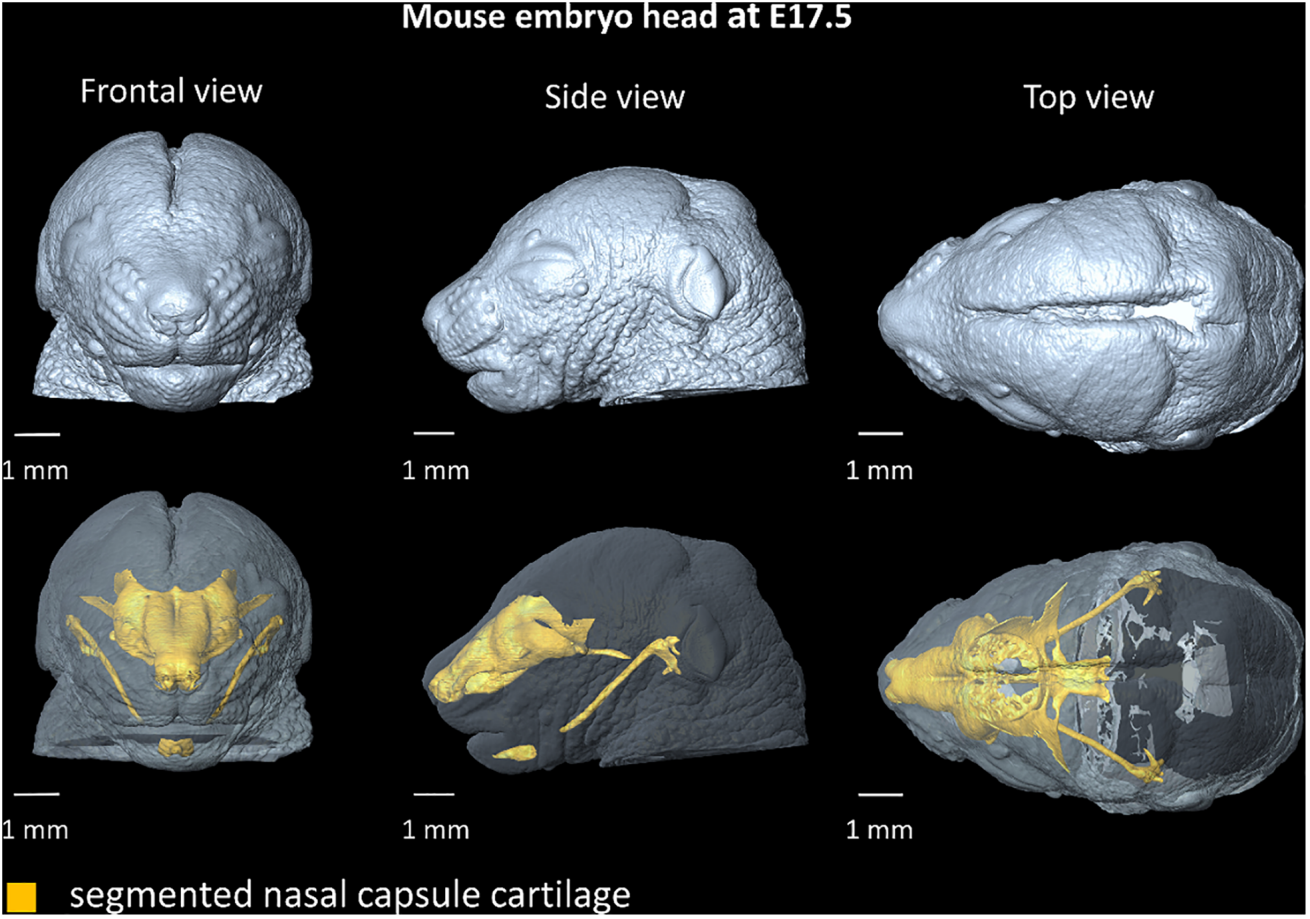
3D reconstruction of a mouse embryo head at E17.5. Yellow 3D model represents segmented nasal capsule and Meckel cartilage in the head.

While μCT has been proven useful for non-destructive high-resolution imaging of high-density biological tissues (e.g., bones [5,6], teeth [7,8]), there are issues with the differentiation between types of soft tissues in the resulting images. The reason is an insufficient difference in their X-ray attenuation coefficients, which results in low contrast in the reconstructed tomographic images [4]. This inherent limitation of absorption-based computed tomography can be addressed by using contrast-enhancing techniques (e.g., staining the sample with contrast-enhancing chemical substances [9]). Several approaches for soft-tissue contrasting are explored in the literature, e.g., osmium tetroxide [10,11], Lugol's iodine [12,13], or phosphotungstic acid (PTA) [4,9]. We used a contrast method based on differential uptake of PTA by various tissues, resulting in excellent resolution and visibility of fine structures (e.g., see tomographic slices in Fig. 1). It enabled us to differentiate between nasal capsule cartilage (and mesenchymal condensations in the images of younger embryos) and surrounding soft tissues. An operator was then able to manually segment the mesenchymal condensations and cartilage forming the nasal capsule of the embryos (Fig. 2). We provide the generated manual segmentations alongside the tomographic slices. These scans can be used by researchers interested in the development of various structures in the head.

The provided atlas of mouse cranial development (including tomographic slices and segmented nasal capsules) will be essential for tracing the normal development of any tissue type within the vertebrate head. Given the excellent differential contrast and general high quality of the data, they can be reused for any investigation of normal anatomy during the developmental time course.

## Methods

### Sample preparation

Mouse embryonic heads were contrasted using PTA-staining procedure, followed by a µCT measurement. The staining protocol is a modification of the protocol pioneered by Metscher [9] and has been described previously [2,14]. Briefly, the mouse embryos were fixed with 4% formaldehyde in phosphate-buffered saline for 24 hours at 4°C. The samples were then washed with phosphate-buffered saline and subsequently dehydrated with 30%, 50%, and 70% ethanol for 1 day each to minimize shrinking of the embryonic tissue. The samples were then transferred into 0.5–1.0% PTA solution (Lach-Ner, Czech Republic) in 90% methanol. The solution was replaced every 2–3 days. The E12.5 sample was left to absorb the contrasting solution for 1 week, the E15.5 for 3 weeks, and the E18.5 for 7 weeks. After stage E15.5, the head was separated from the body at the level of the shoulders to ensure an adequate and uniform contrasting. After this staining procedure was completed, the samples were rehydrated in a methanol series of decreasing concentration (90%, 70%, 50%, and 30%). Prior to the μCT scanning, the samples were submerged in 0.5% agarose gel (A5304, Sigma-Aldrich, St. Louis, MO, USA)) and placed in polypropylene conical tubes with volume ranging from 0.5 to 15 mL. The tube volume was chosen with respect to the size of the sample to obtain images of the best possible quality. To further characterize the embryonal stages in addition to their age after fertilization, Theiler staging was performed [15]. The prepared samples are listed in Table 1. The *Mus musculus* C57BL/6NCrl samples were sourced from Charles River Laboratories, Germany (IMSR Cat# CRL_27, RRID:IMSR_CRL:27).

**Table 1:** List of *Mus musculus* C57BL/6NCrl samples

Resource	Age (days)	Theiler stage	Voxel size (μm)
Embryo 1	12.5	TS 20	2.6
Embryo 2	13.5	TS 21	3.0
Embryo 3	14.5	TS 23	5.0
Embryo 4	15.5	TS 24	6.0
Embryo 5	16.5	TS 25	6.0
Embryo 6	17.5	TS 26	5.8
Embryo 7	18.5	TS 26	5.5

### Image acquisition

The samples were scanned with a laboratory-based μCT system, GE Phoenix v|tome|x L 240 (GE Sensing & Inspection Technologies GmbH, Germany). The system was equipped with a high-contrast flat-panel detector DXR250 with 2,048 × 2,048 pixel resolution. The embryos were fixed in polypropylene conical tubes with 0.5% agarose gel to prevent sample movement during the µCT stage rotation. A total of 2,000 projections were acquired with an exposure time of 900 ms per projection. Each projection was captured 3 times, and an average of the signal was used to improve the signal-to-noise ratio. The acceleration voltage of the X-ray tube was 60 kV, and the tube current, 200 μA. The X-ray beam was filtered with a 0.1-mm aluminium plate. The time required for scanning 1 sample was 1 hour and 30 minutes.

### Software processing

Tomographic reconstruction of the obtained set of projections was performed with GE phoenix datos |x 2.0 3D computed tomography software (GE Sensing & Inspection Technologies GmbH, Germany), which allowed a 3D image of the mouse embryo head to be generated. The voxels are isotropic; voxel sizes for individual samples are listed in Table 1.

### Manual segmentation

Avizo (Thermo Fisher Scientific, Waltham, MA, USA) image-processing software was used for manual segmentation of the mesenchymal condensations and nasal capsule cartilage in the reconstructed CT images. Avizo is a commercial software package providing a broad range of tools for manipulating and processing 3D image data. The manual segmentation of the cartilaginous nasal capsule tissue takes ∼10–20 hours depending on the size of the sample and the experience of the operator. To make the load of 3D segmentation volume smaller, only every third slice was manually segmented and the rest was calculated by linear interpolation between adjacent manually segmented slices [14]. This 3-fold increase in segmentation speed does not significantly affect the accuracy of the segmentation because the small slice width keeps differences in structures in adjacent slices to a minimum. The overlap between the segmentation performed on a slice-by-slice basis and segmentation with interslice interpolation is >98% (Dice coefficient) in the case of this type of sample. The cartilage was segmented in 2D slices of the whole 3D volume, so there is in some cases a staircase artefact present in the planes other than the plane in which the segmentation was performed.

### Data validation and quality control

**Figure 3:**
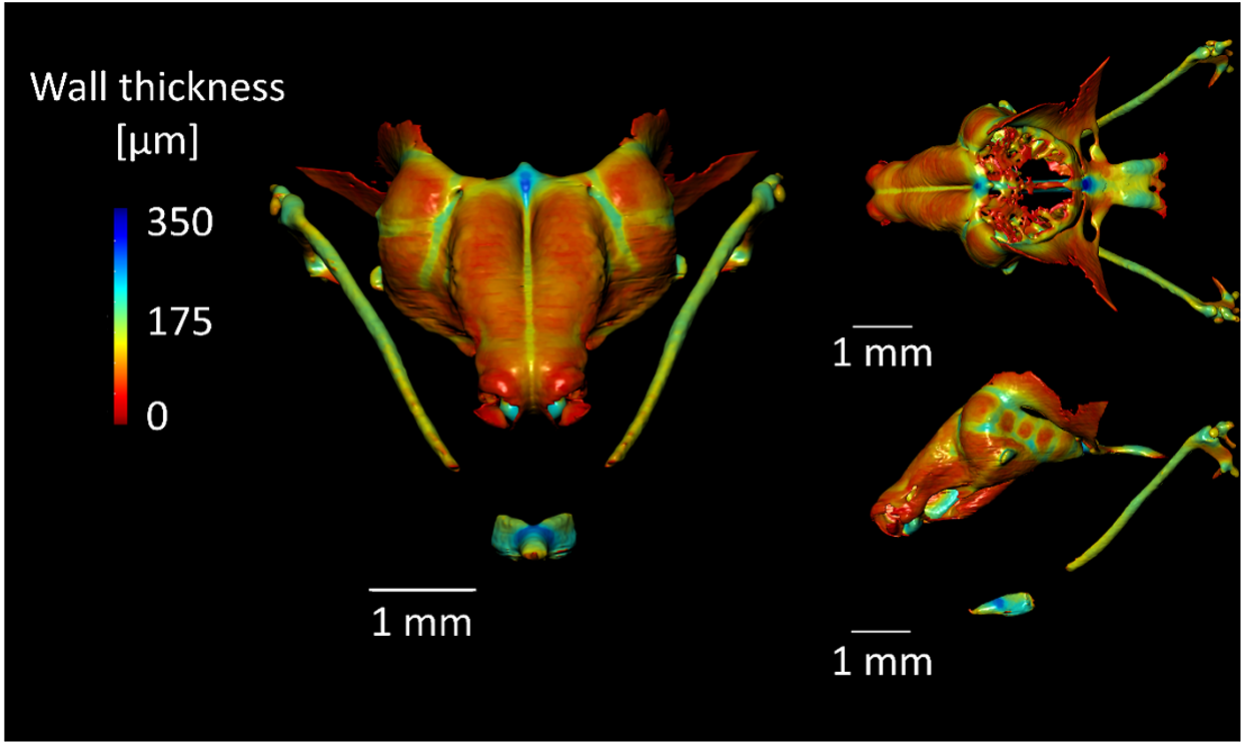
Wall thickness analysis of the manually segmented mouse embryonic nasal capsule (sample E17.5). The wall thickness is calculated as the diameter of a hypothetical sphere that fits within boundary points of the nasal capsule mesh. The 3D wall thickness model was created in the Dragonfly software (Object Research Systems [ORS] Inc., Canada).

The segmented 3D models of nasal capsule can be subjected to various subsequent analyses that further highlight the differences between compared models from distinct samples. For instance, wall thickness analysis of the segmented nasal capsule provides valuable information outside of the general morphology assessment of the mouse embryonic anterior face. This information serves to compare multiple samples and provides quantitative information on the variability within each specimen (Fig. 3). Such an approach was instrumental in the work of Kaucka and collaborators [1,2], where the wall thickness analysis was used to dissect the fundamental mechanisms of cartilage growth and highlighted the molecular basis of the thickness regulation. The obtained results were implemented in a mathematical model that could predict the underlying cellular dynamics of the cartilage growth. Furthermore, using this method it was possible to depict subtle differences between control and mutant embryonic samples that seemed otherwise morphologically similar [1]. Together with core measurements such as the width and the length (see Table 3) of the nasal capsule and mapping the surface expansion during embryonic growth, the authors acquired a detailed understanding of the shaping and the growth of this complex structure.

**Table 3:** Length and width measurement of the manually segmented nasal capsule performed in the Avizo (Thermo Fisher Scientific, USA) software with the Measure tool

Sample	Length (mm)	Width (mm)
Embryo 1	0.48	1.37
Embryo 2	0.90	1.41
Embryo 3	1.33	1.53
Embryo 4	1.56	1.99
Embryo 5	2.12	2.53
Embryo 6	2.34	2.83
Embryo 7	2.56	2.85

**Figure 4:**
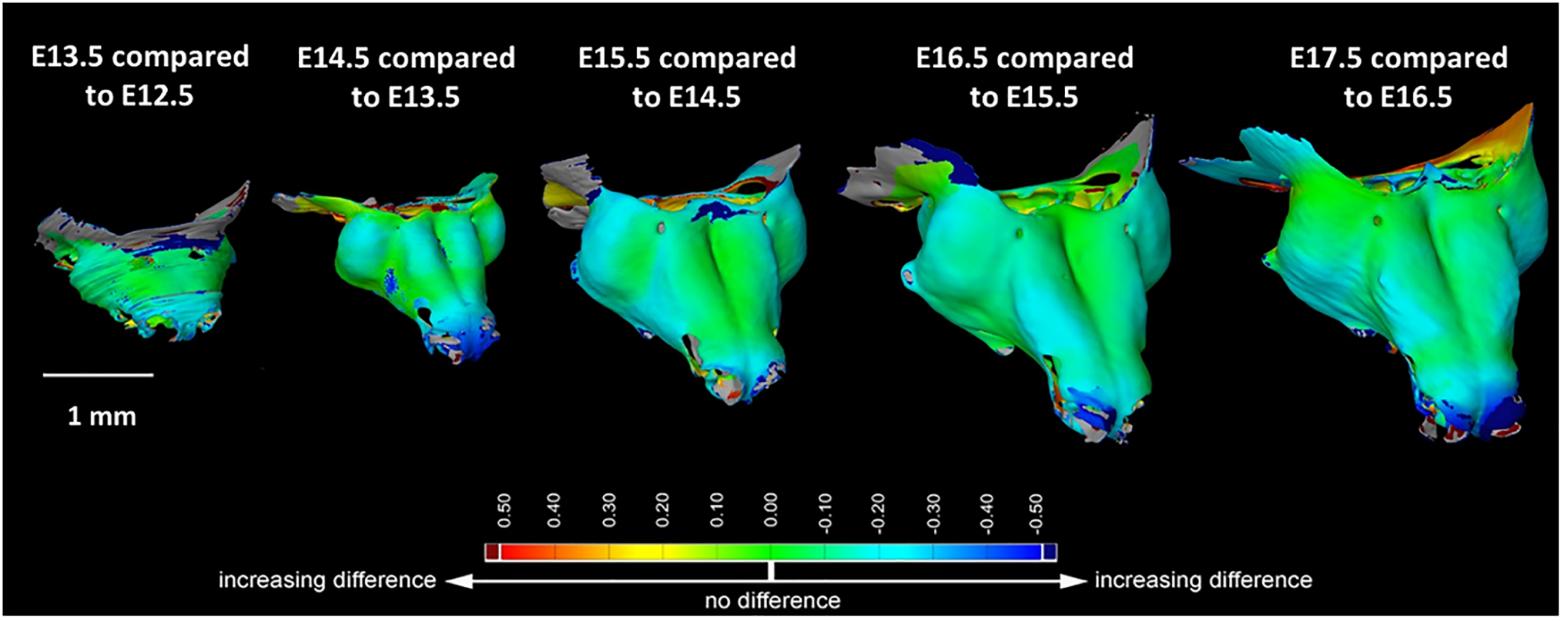
Manually segmented nasal capsules of developmental stages E13.5, E14.5, E15.5, E16.5, and E17.5 were compared to the previous developmental stage in the GOM Inspect software. Figure adapted from Fig. 3—figure supplement 2 from [1] under CC BY 4.0.

Shape comparison between individual stages of development provides us with valuable information about the areas of the sample where growth is the most prominent. Figure 4 depicts such an analysis performed on the nasal capsule of embryos in developmental stages ranging from 12.5 to 17.5 days old [1]. This analysis was done in the software GOM Inspect (GOM GmbH, Germany).

**Figure 5:**
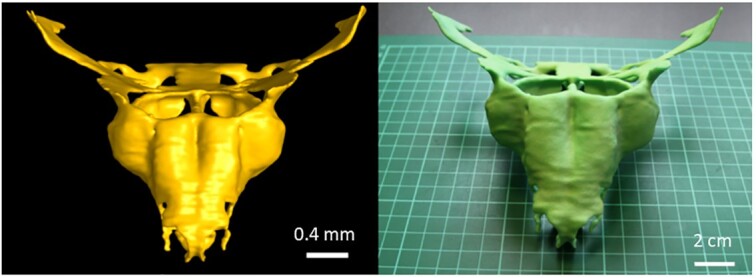
3D-printed model of the mouse embryo nasal capsule (right) next to its 3D render created from manually segmented binary masks (left). Figure adapted from Fig. 7 from [14] under CC BY 3.0.

By manually segmenting the nasal capsule cartilage in reconstructed images of the samples, we were able to obtain an anatomically accurate 3D-printed model of the embryonic mouse nasal capsule. This is beneficial for researchers to physically evaluate the morphology of the embryonic head. Precise visualization of the developing nasal capsule, together with the opportunity to produce a physical 3D-printed model of this complex anatomical structure, allows better understanding of the organization of single skeletal elements in the framework of the sophisticated organization of the mammalian embryonic head [14] (Fig. 5).

### Reuse potential

This dataset with its high-quality manually segmented masks can be instrumental in creating a robust method for segmentation of cartilaginous structures from μCT images of mouse embryos. The field of image processing has lately been dominated by deep learning algorithms, and specifically convolutional neural networks consistently achieve state-of-the-art results in fully automatic image segmentation tasks [16]. A segmentation model created in such way could make acquiring new samples for analysis of nasal capsule development in mouse embryos much less time consuming because the time-expensive process of manual segmentation would be eliminated. Nevertheless, high-quality scans with a sufficient tissue contrast are required for such automated segmentation. Our dataset has been validated for its suitability for such deep learning algorithm application and can be therefore used by other researchers for this purpose as well.

### Biological potential

The possibilities to reuse this dataset are broad and include the analysis of developmental changes in nasal epithelium, eyes, whiskers, tongue, oral cavity, developing teeth, brain, cranial cartilage and bone, tendons, muscles, endocrine organs, vessels, and nerves. For instance, questions pertaining to the mechanisms controlling the growth and shaping of the brain or craniofacial skeleton are still open and will benefit from the presented data. Furthermore, during development and growth, multiple tissue interactions and integration events occur at multiple morphologically distinct tissue interfaces. Such interactions at the tissue scale lead to the development of muscle attachments, correct vascularization, innervation, and many other key developmental events. This dataset embraces late stages of mouse cranial development when the definitive tissue integration events take place. Without doubt, such tomographic data will be suitable for improving our understanding of these fundamental questions.

## Data Availability

The data underlying this article are available in the *GigaScience* Database repository [17]. We provide already-reconstructed μCT data. The dataset is presented as 8-bit TIFF stacks of corresponding computed tomographic slices and manually segmented masks. The folders are structured so that each folder representing 1 sample contains 2 folders: Images and Masks. The Images folder contains reconstructed tomographic slices in TIFF format, and the folder Masks contains corresponding manually segmented masks. The naming convention is as follows: Sample_name.tif for slice and mask_Sample_name.tif for segmented mask. To enable the users of the dataset to visually inspect the embryo heads in 3 dimensions, .stl files were included together with the image stacks. In addition, a text file is provided for each sample containing information about the voxel size.

As TIFF stacks, the deposited data can be opened and viewed in any basic image viewer; however, to fully take advantage of the possibilities provided by the 3D nature of the images, a specialized viewer for 3D data is recommended. Avizo (Thermo Fisher Scientific, Waltham, MA, USA) is a commercial software package providing a broad range of possibilities to visualize, manipulate, and analyse 3D μCT image data. Another commercial software option is VG Studio MAX (Volume Graphics GmbH, Germany). We recommend the Fiji ImageJ distribution [18] as a free software option to view and manipulate the provided data. Because the manually segmented masks of the data are binary images composed of 0s (background) and 1s (mesenchymal condensations/cartilage), they can be displayed as black images. To visually inspect the data, it may be necessary to set the software display window to the range from 0 to 1 in some image viewers. The included .stl files of the embryo heads can be explored in many different 3D mesh viewers; a popular free open-source software option is MeshLab [19]. Reconstructions are also available for browsing in Sketchfab [20].

## Abbreviations

μCT: X-ray microtomography; GE: General Electric; PTA: phosphotungstic acid.

## Ethics Approval and Consent to Participate

All animal work was approved and permitted by the Local Ethical Committee on Animal Experiments (North Stockholm Animal Ethics Committee) and conducted according to The Swedish Animal Agency´s Provisions and Guidelines for Animal Experimentation recommendations.

## Competing Interests

The authors declare that they have no competing interests.

## Funding

This research was carried out under the project CEITEC 2020 (LQ1601) with financial support from the Ministry of Education, Youth and Sports of the Czech Republic under the National Sustainability Programme II and CzechNanoLab Research Infrastructure supported by MEYS CR (LM2018110). J.M. was financially supported by grant CEITEC VUT-J-20–6477. M.T. was financially supported by grant CEITEC VUT-J-20–6317 and by the Brno City Municipality as a Brno Ph.D. Talent Scholarship Holder.

## Authors' Contributions

J.M.: Conceptualization, writing—original draft, visualization, writing—review and editing. M.T.: Methodology, data curation, writing—review and editing. T.Z.: Conceptualization, writing—review and editing. M.K.: Writing—original draft, writing—review and editing. I.A.: Writing—original draft. J.K.: Funding acquisition, supervision, project administration.

## Supplementary Material

giab012_GIGA-D-20-00282_Original_Submission

giab012_GIGA-D-20-00282_Revision_1

giab012_Response_to_Reviewer_Comments_Original_Submission

giab012_Reviewer_1_Report_Original_Submission Brian Metscher -- 10/10/2020 Reviewed

giab012_Reviewer_2_Report_Original_Submission Jaesung Peter Choi, Ph.D -- 11/3/2020 Reviewed

giab012_Reviewer_2_Report_Revision_1 Jaesung Peter Choi, Ph.D -- 1/11/2021 Reviewed

giab012_Reviewer_3_Report_Original_Submission Chris Armit -- 11/3/2020 Reviewed

giab012_Reviewer_3_Report_Revision_1 Chris Armit -- 1/13/2021 Reviewed
