# Supplementary material for: X-ray microtomography–based atlas of mouse cranial development
Source: Gigascience. 2021 Mar 2;10(3):giab012. doi: 10.1093/gigascience/giab012 (PMC7936920; doi:10.1093/gigascience/giab012)

GigaScience

Micro X-ray computed tomography-based atlas of mouse cranial development

--Manuscript Draft--

**Manuscript Number:**	GIGA-D-20-00282
**Full Title:**	Micro X-ray computed tomography-based atlas of mouse cranial development
**Article Type:**	Data Note
**Funding Information:**	Ministerstvo Školství, Mládeže a Tělovýchovy (LQ1601/LM2018110) — Not applicable<br>Vysoké Učení Technické v Brně (CEITEC VUT-J-20-6477) — Mr. Jan Matula<br>Vysoké Učení Technické v Brně (CEITEC VUT-J-20-6317) — Mrs. Marketa Tesarova
**Abstract:**	Background<br><br>Micro X-ray computed tomography (μCT) has become an invaluable tool for non-destructive analysis of biological samples in the field of developmental biology. Mouse embryos are typical model for investigation of human developmental diseases. By obtaining 3-D high resolution scans of the mouse embryo heads, we gain valuable morphological information about the structures prominent in the development of future face, brain and sensory organs. The development of facial skeleton tracked in these μCT data provides a valuable background for further studies of congenital craniofacial pathologies.<br><br>Findings<br><br>In this work, re-usable tomographic data from 7 full 3-D scans of mouse embryo heads are presented and made publicly available. The ages of these embryos range from E12.5 to E18.5. The samples were stained by phosphotungstic acid prior to scanning, which greatly enhanced the contrast of various tissues in the reconstructed images and enabled precise segmentation. The images were obtained on a lab-based μCT system. Furthermore, we provide manually segmented masks of mesenchymal condensations (for E12.5 and E13.5) and cartilage present in the nasal capsule of the scanned embryos.<br><br>Conclusion<br><br>We present a comprehensive dataset of X-ray 3-D computed tomography images of the developing mouse head with high-quality manual segmentation masks of cartilaginous nasal capsules. The provided μCT images can be used for studying any other major structure within the developing mouse heads. The high quality of the manually segmented models of nasal capsules may be instrumental to understand the complex process of the development of the face in mouse model.
**Corresponding Author:**	Tomas Zikmund, PhD<br>Central European Institute of Technology, Brno University of Technology<br>Brno, CZECH REPUBLIC
**Corresponding Author Secondary Information:**	
**Corresponding Author's Institution:**	Central European Institute of Technology, Brno University of Technology
**Corresponding Author's Secondary Institution:**	
**First Author:**	Jan Matula, MSc
**First Author Secondary Information:**	
**Order of Authors:**	Jan Matula, MSc<br>Marketa Tesarova, MSc<br>Tomas Zikmund, PhD

Powered by Editorial Manager® and ProduXion Manager® from Aries Systems Corporation

	Marketa Kaucka, PhD
	Igor Adameyko, PhD
	Jozef Kaiser, PhD
**Order of Authors Secondary Information:**	
**Additional Information:**	
**Question**	**Response**
Are you submitting this manuscript to a special series or article collection?	No
**Experimental design and statistics**<br><br>Full details of the experimental design and statistical methods used should be given in the Methods section, as detailed in our Minimum Standards Reporting Checklist. Information essential to interpreting the data presented should be made available in the figure legends.<br><br>Have you included all the information requested in your manuscript?	Yes
**Resources**<br><br>A description of all resources used, including antibodies, cell lines, animals and software tools, with enough information to allow them to be uniquely identified, should be included in the Methods section. Authors are strongly encouraged to cite Research Resource Identifiers (RRIDs) for antibodies, model organisms and tools, where possible.<br><br>Have you included the information requested as detailed in our Minimum Standards Reporting Checklist?	Yes
**Availability of data and materials**<br><br>All datasets and code on which the conclusions of the paper rely must be either included in your submission or deposited in publicly available repositories (where available and ethically	Yes

appropriate), referencing such data using a unique identifier in the references and in the “Availability of Data and Materials” section of your manuscript.<br><br>Have you have met the above requirement as detailed in our Minimum Standards Reporting Checklist?	

Powered by Editorial Manager® and ProduXion Manager® from Aries Systems Corporation

Manuscript
Click here to access/download;Manuscript;data_note_JM_final_rev.docx

Micro X-ray computed tomography-based atlas of mouse cranial development

J. Matula[1], M. Tesarova[1], T. Zikmund[1], M. Kaucka[2,3], I. Adameyko[3], J. Kaiser[1]

[1] Central European Institute of Technology, Brno University of Technology, Brno, Czech Republic

[2] Max Planck Institute for Evolutionary Biology, Plön, Germany

[3] Medical University Vienna, Vienna, Austria

Abstract

Background: Micro X-ray computed tomography (μCT) has become an invaluable tool for non-destructive analysis of biological samples in the field of developmental biology. Mouse embryos are typical model for investigation of human developmental diseases. By obtaining 3-D high resolution scans of the mouse embryo heads, we gain valuable morphological information about the structures prominent in the development of future face, brain and sensory organs. The development of facial skeleton tracked in these μCT data provides a valuable background for further studies of congenital craniofacial pathologies.

Findings: In this work, re-usable tomographic data from 7 full 3-D scans of mouse embryo heads are presented and made publicly available. The ages of these embryos range from E12.5 to E18.5. The samples were stained by phosphotungstic acid prior to scanning, which greatly enhanced the contrast of various tissues in the reconstructed images and enabled precise segmentation. The images were obtained on a lab-based μCT system. Furthermore, we provide manually segmented masks of mesenchymal condensations (for E12.5 and E13.5) and cartilage present in the nasal capsule of the scanned embryos.

Conclusion: We present a comprehensive dataset of X-ray 3-D computed tomography images of the developing mouse head with high-quality manual segmentation masks of cartilaginous nasal capsules. The provided µCT images can be used for studying any other major structure within the developing mouse heads. The high quality of the manually segmented models of nasal capsules may be instrumental to understand the complex process of the development of the face in mouse model.

Keywords

X-ray, computed tomography, mouse embryo head, tissue contrast, 3-D modelling, nasal capsule

Data description

Context

Vertebrate head is considered one of the most complex parts of the body. The head is formed during the embryonic development through a process known as morphogenesis, which involves hundreds of genes and non-coding regulatory sequences [1,2]. This intricate body compartment hosts numerous cell and tissue types forming, for instance, muscles, ligaments, nerves and central nervous system, sensory organs, hair follicles, teeth, which are all integrated in the complexly shaped skull. There is a remarkable inter- but in some cases (such as humans) also intra-species variability of the craniofacial shapes [3]. Reportedly, the shape of the face (or the whole head) depends on the geometry of the skeleton that provides protection to sensitive nervous tissues and serves as a scaffold for muscle attachment [1]. The skeleton of the head is formed by two types of stiff tissue – bone and cartilage. Although the majority of head skeleton in mammals is formed by bones postnatally, the embryonic development of the skull relies on the cartilage. Chondrocranium is induced as 14 independent pieces that grow, acquire specific shape and fuse later on to form the skull [1]. Interestingly, the development of cartilage and bone corresponds to the progress of development of central and peripheral nervous system and sensory organs [2]. Therefore, what is the exact developmental link between the emergence of nervous structures and the appearance of cartilage and bone, is one of the fundamental

questions in developmental biology. At the same time, understanding both molecular basis and cellular dynamics driving the formation and shaping of the mammalian head is of utmost interest in the field of clinical genetics and regenerative medicine, dealing with a broad spectrum of human congenital craniofacial disorders.

In our previous work, we aimed to explore the exact sequence of formation and shaping of the mammalian developing face and we used a mouse model for our investigation [1,2]. The morphological properties of the observed structures are complex and to fully understand their shaping, advanced imaging techniques are required. X-ray computed tomography technique is one of the oldest imaging techniques, but in recent years it has shown its strengths in the field of developmental biology [4]. The principle of X-ray computed tomography lies at acquiring 2-D projections of the scanned sample at regular angle increments. 3-D view of the scene is then created by the process of tomographic reconstruction. This way we gain 3-D spatial information that would be otherwise unobtainable without destroying the sample. The superior resolution of modern lab-based μCT machines provides a way to visualize and analyze biological structures on the level of units of micrometres and more importantly, in the 3-D spatial context. We combined genetic tracing, gene knock-out strategies, mathematical modelling and μCT to reconstruct the craniofacial development in detail. As a result, we generated a full set of μCT scans from wild type mouse strains, ranging from E12.5 (where the first induction of early cartilage, represented by condensation of the mesenchyme, can be observed) to E18.5 with fully formed chondrocranium.

While μCT has been proven useful for non-destructive high-resolution imaging of high-density biological tissues (e.g. bones [5,6], teeth [7,8]), there are issues with the differentiation between types of soft tissues in the resulting images. The reason is an insufficient difference in their X-ray attenuation coefficients, which results in low contrast in the reconstructed tomographic images [4]. This inherent limitation of absorption-based computed tomography can be addressed by utilizing contrast-enhancing techniques (e.g. staining the sample with contrast-enhancing chemical substances [9]). We

used a tissue-contrasting method based on a different uptake of phosphotungstic acid (PTA) by various tissues, resulting in an unprecedented resolution and visibility of fine structures (see Figure 1 for example tomographic slices). It enabled us to differentiate between nasal capsule cartilage (and mesenchymal condensations in the images of younger embryos) and surrounding soft tissues. An operator was then able to manually segment the mesenchymal condensations and cartilage forming the nasal capsule of the embryos (Figure 2). We provide the generated manual segmentations alongside the tomographic slices. These scans can be used by researchers interested in the development of various structures in the head.

Figure 1: Examples of tomographic slices of mouse embryos 12.5 days old (E12.5) and 18.5 days old (E18.5). µCT scanning of samples stained with PTA provides image data with excellent contrast, where even fine details are visible. Yellow arrows show areas of the imaged that might be interesting for potential users of the provided dataset.

Figure 2: 3-D reconstruction of a mouse embryo head E17.5. Yellow 3-D model represents segmented nasal capsule and Meckel cartilage in the head.

The provided atlas of mouse cranial development (including tomographic slices and segmented nasal capsules) will be essential for tracing normal development of any tissue type within the vertebrate head. Given the excellent differential contrast and general high quality of the data, they can be re-used for any investigation of normal anatomy during developmental time course.

Methods

Sample preparation

Mouse embryonic heads were contrasted using PTA-staining procedure, followed by a µCT measurement. The staining protocol is a modification of the protocol pioneered by Brian Metscher [9] and has been described previously in [2,10]. Briefly, the mouse embryos were fixed with 4% formaldehyde in phosphate buffer saline (PBS) for 24 hours at 4 °C. The samples were then washed with PBS and subsequently dehydrated with 30%, 50% and 70% ethanol for 1 day each to minimize shrinking of the embryonic tissue. The samples were then transferred into 1.0-1.5% PTA solution in

90% methanol. The solution was replaced every 2 to 3 days. The E12.5 sample was left to absorb the contrasting solution for 7 days, the E15.5 for 21 days and the E18.5 for 49 days. Starting with the stage E15.5, the head was separated from the body at the level of shoulders to ensure an adequate and uniform contrasting. After this staining procedure was completed, the samples were rehydrated in ethanol series of decreasing concentration (90 %, 70 %, 50 % and 30 %). Prior to the µCT scanning, the samples were submerged in 1% agarose gel and placed in polypropylene conical tubes with volume ranging from 0.5 to 15 ml. The tube volume was chosen with respect to the size of the sample in order obtain images of the best possible quality. The prepared samples are listed in Table 1.

Table 1: List of samples

Resource	Organism	Strain	Age	Source	RRID
Embryo 1	Mus musculus	C57BL/6NCrl	12.5 days after fertilization	Charles River Germany	RRID:IMSR_CRL:27
Embryo 2	Mus musculus	C57BL/6NCrl	13.5 days after fertilization	Charles River Germany	RRID:IMSR_CRL:27
Embryo 3	Mus musculus	C57BL/6NCrl	14.5 days after fertilization	Charles River Germany	RRID:IMSR_CRL:27
Embryo 4	Mus musculus	C57BL/6NCrl	15.5 days after fertilization	Charles River Germany	RRID:IMSR_CRL:27
Embryo 5	Mus musculus	C57BL/6NCrl	16.5 days after fertilization	Charles River Germany	RRID:IMSR_CRL:27

Embryo 6	Mus musculus	C57BL/6NCrl	17.5 days after fertilization	Charles River Germany	RRID:IMSR_CRL:27
Embryo 7	Mus musculus	C57BL/6NCrl	18.5 days after fertilization	Charles River Germany	RRID:IMSR_CRL:27

Image acquisition

The samples were scanned with a lab-based µCT system GE Phoenix v|tome|x L 240 (GE Sensing & Inspection Technologies GmbH Germany). The system was equipped with a high contrast flat panel detector DXR250 with 2048 × 2048 pixel resolution. The embryos were fixed in polyimide tubes filled with 1% agarose gel to prevent the sample movement during the µCT stage rotation. 2000 projections were acquired with an exposure time of 900 ms per projection. Each projection was captured three times and an average of the signal was used to improve the signal-to-noise ratio. The acceleration voltage of the X-ray tube was 60 kV and the tube current 200 µA. The X-ray beam was filtered with a 0.1 mm aluminium plate.

Software processing

Tomographic reconstruction of the obtained set of projections was performed with GE phoenix datos |x 2.0 3-D computed tomography software (GE Sensing & Inspection Technologies GmbH Germany), which allowed to generate a 3-D image of the mouse embryo head. The voxels are isotropic, the voxel sizes for individual samples are shown in Table 2.

Table 2: Voxel sizes of individual samples

Sample	Voxel size [µm]
Embryo 1	3.2
Embryo 2	3

Embryo 3	5
Embryo 4	6
Embryo 5	6
Embryo 6	5.8
Embryo 7	5.5

Manual segmentation

Avizo (Thermo Fisher Scientific, USA) image processing software was used for manual segmentation of the mesenchymal condensations and nasal capsule cartilage in the reconstructed CT images. Avizo is a commercial software providing a broad range of tools for manipulating and procession 3-D image data. The manual segmentation of the cartilaginous nasal capsule tissue takes approximately 10 to 20 hours depending on the size of the sample and the experience of the operator. To make the load of 3-D segmentation volume smaller, only every 3rd slice was manually segmented and the rest was calculated by linear interpolation between adjacent manually segmented slices [10]. This three-fold increase in segmentation speed does not affect the accuracy of the segmentation in a significant way, because the small slice width makes differences in structures in adjacent slices minimal. The cartilage was segmented in 2-D slices of the whole 3-D volume, so there is in some cases a staircase artefact present in the planes other than the plane, in which the segmentation was performed.

Data validation and quality control

The segmented 3-D models of nasal capsule can be subjected to various subsequent analyses that further highlight the differences between compared models from distinct samples. For instance, wall thickness analysis of the segmented nasal capsule provides valuable information outside of the general morphology assessment of the mouse embryonic anterior face. This information serves to compare multiple samples and provides quantitative information on the variability within the samples (Figure 3). Such an approach was instrumental in the work of Kaucka and collaborators [1,2] where

the wall thickness analysis was used to dissect the fundamental mechanisms of cartilage growth and highlighted the molecular basis of the thickness regulation. The obtained results were implemented in the mathematical model that could predict the underlying cellular dynamics of the cartilage growth. Furthermore, using this method it was possible to depict subtle differences between control and mutant embryonic samples that appeared otherwise morphologically similar [1]. Together with core measurements such as the width and the length of the nasal capsule and mapping the surface expansion during the embryonic growth, authors acquired detailed understanding of the shaping and the growth of this complex structure.

Figure 3: Wall thickness analysis of the manually segmented mouse embryonic nasal capsule (sample E17.5). The wall thickness is calculated as the diameter of a hypothetical sphere that fits within boundary points of the nasal capsule mesh. The 3-D wall thickness model was created in the Dragonfly software (Object Research Systems (ORS) Inc., Canada).

Shape comparison between individual stages of development provides us with valuable information about the areas of the sample, where growth is the most prominent. Figure 4 depicts such analysis performed on nasal capsule of embryos in developmental stages ranging from 12.5 to 17.5 days old [1]. This analysis was done in the software GOM Inspect (GOM GmbH, Germany).

Figure 4: Manually segmented nasal capsules of developmental stages E13.5, E14.5, E15.5, E16.5 and E17.5 were compared to the previous developmental stage in the GOM Inspect Software. Figure adapted from Figure 3—figure supplement 2 from [1] *under CC BY 4.0.*

By manually segmenting the nasal capsule cartilage in reconstructed images of the samples, we were able to obtain anatomically accurate 3-D printed model of the embryonic mouse nasal capsule. This is very beneficial for researchers to physically evaluate the morphology of the embryonic head. Precise visualisation of the developing nasal capsule together with the opportunity to produce a physical 3-D-printed model of this complex anatomical structure allows better understanding of the organization of single skeletal elements in the framework of the sophisticated organisation of mammalian embryonic head [10]. (Figure 5).

Figure 5: 3-D printed model of the mouse embryo nasal capsule (right) next to its 3-D render created from manually segmented binary masks (left). Figure adapted from Figure 7 from [10] *under CC BY 3.0.*

Re-use potential

This dataset with its high quality manually segmented masks can be instrumental in creating a robust method for segmentation of cartilaginous structures from µCT images of mouse embryos. The field of image processing is lately being dominated by deep learning algorithms and specifically convolutional neural networks (CNNs) consistently achieve state-of-the-art results in fully-automatic image segmentation tasks [11]. A segmentation model created in such way could make acquiring new samples for analysis of nasal capsule development in mouse embryos much less time consuming, because the time-expensive process of manual segmentation would be eliminated. Nevertheless, high quality scans with a sufficient tissue contrast are required for such automated segmentation. Our dataset has been validated for its suitability for such deep learning algorithm application and can be therefore used by other researchers for this purpose as well.

Biological potential:

The possibilities to re-use this dataset are broad and include the analysis of developmental changes in nasal epithelium, eyes, whiskers, tongue, oral cavity, developing teeth, brain, cranial cartilage and bone, tendons, muscles, endocrine organs, vessels and nerves. For instance, questions pertaining to the mechanisms controlling growth and shaping of the brain or craniofacial skeleton are still open, and will benefit from presented data. Furthermore, during development and growth, multiple tissue-interactions and integration events occur at multiple morphologically distinct tissue interfaces. Such interactions at the tissue scale lead to the development of muscle attachments, correct vascularization, innervation and many other key developmental events. This dataset embraces late stages of mouse cranial development when the definitive tissue integration events take place. Without doubts, such tomographic data will be suitable for improving our understanding of these fundamental questions.

Availability of supporting data

The dataset presented in this work is available through the GigaScience Database repository. We provide already reconstructed X-ray computed tomography data. The dataset is presented as 8-bit tiff stacks of corresponding CT slices and manually segmented masks. The folders are structured in a way, that each folder representing one sample contains two folders: Images and Masks. The Images folder contains reconstructed tomographic slices in Tiff format, the folder Masks contains corresponding manually segmented masks. The naming convention is: Sample_name.tif for slice and mask_Sample_name.tif for segmented mask. Additionally, a text file is provided for each sample containing information about the voxel size.

As tiff stacks, the deposited data can be opened and viewed in any basic image viewer, however, to fully take advantage of the possibilities provided by the 3-dimensional nature of the images, a specialized viewer for 3-D data is recommended. Avizo (Thermo Fisher Scientific, USA) is a commercial software providing a broad range of possibilities to visualize, manipulate and analyze 3-D µCT image data. Another commercial software option is VG Studio MAX (Volume Graphics GmbH, Germany). We recommend the Fiji ImageJ distribution [12] as a free software option to view and manipulate the provided data.

Declarations

List of abbreviations

µCT	micro X-ray computed tomography
CNN	convolutional neural network
GE	General Electric
PTA	phosphotungstic acid
VG	Volume Graphics

Ethics approval and consent to participate

All animal work was approved and permitted by the Local Ethical Committee on Animal Experiments (North Stockholm Animal Ethics Committee) and conducted according to The Swedish Animal Agency´s Provisions and Guidelines for Animal Experimentation recommendations.

Consent for publication

Not applicable.

Competing interests

The authors declare no competing interests.

Funding

This research was carried out under the project CEITEC 2020 (LQ1601) with financial support from the Ministry of Education, Youth and Sports of the Czech Republic under the National Sustainability Programme II and CzechNanoLab Research Infrastructure supported by MEYS CR (LM2018110).

JM was financially supported by grant CEITEC VUT-J-20-6477.

MT was financially supported by grant CEITEC VUT-J-20-6317 and by the Brno City Municipality as a Brno Ph.D. Talent Scholarship Holder.

Authors' contributions

JM	conceptualization, writing – original draft, visualization
MT	methodology, data curation, writing – review & editing
TZ	conceptualization, writing – review & editing
MK	writing – original draft, writing – review & editing
IA	writing – original draft
JK	funding acquisition, supervision, project administration

Acknowledgements

Not applicable.

References

1. Kaucka M, Zikmund T, Tesarova M, Gyllborg D, Hellander A, Jaros J, et al. Oriented clonal cell dynamics enables accurate growth and shaping of vertebrate cartilage. Elife. 2017; doi:10.7554/eLife.25902.

2. Kaucka M, Petersen J, Tesarova M, Szarowska B, Kastriti ME, Xie M, et al. Signals from the brain and olfactory epithelium control shaping of the mammalian nasal capsule cartilage. Elife. 2018; doi:10.7554/eLife.34465.

3. Zollikofer CPE, Ponce de León MS. Visualizing patterns of craniofacial shape variation in Homo sapiens. Proc R Soc B Biol Sci. 2002; doi:10.1098/rspb.2002.1960.

4. Metscher BD. MicroCT for developmental biology: A versatile tool for high-contrast 3D imaging at histological resolutions. Dev Dyn. 2009; doi:10.1002/dvdy.21857.

5. Balto K, Muller R, Carrington DC, Dobeck J, Stashenko P. Quantification of periapical bone destruction in mice by micro-computed tomography. J Dent Res. 2000; doi:10.1177/00220345000790010401.

6. Sabolova V, Brinek A, Sladek V. The effect of hydrochloric acid on microstructure of porcine (Sus scrofa domesticus) cortical bone tissue. Forensic Sci Int. 2018; doi:10.1016/j.forsciint.2018.08.030.

7. Dosedelova H, Stepankova K, Zikmund T, Lesot H, Kaiser J, Novotny K, et al. Age-related changes in the tooth–bone interface area of acrodont dentition in the chameleon. J Anat. 2016; doi: 10.1111/joa.12490.

8. Landova Sulcova M, Zahradnicek O, Dumkova J, Dosedelova H, Krivanek J, Hampl M, et al.

Developmental mechanisms driving complex tooth shape in reptiles. Dev Dyn. 2020; doi:10.1002/dvdy.138.

9. Metscher BD. Micro CT for comparative morphology: Simple staining methods allow high-contrast 3D imaging of diverse non-mineralized animal tissues. BMC Physiol. 2009; doi:10.1186/1472-6793-9-11.

10. Tesarova M, Zikmund T, Kaucka M, Adameyko I, Jaros J, Palousek D, et al. Use of micro computed-tomography and 3D printing for reverse engineering of mouse embryo nasal capsule. J Instrum. 2016; doi:10.1088/1748-0221/11/03/C03006.

11. Ronneberger O, Fischer P, Brox T. U-net: Convolutional networks for biomedical image segmentation. Medical Image Computing and Computer-Assisted Intervention - MICCAI 2015. 2015; doi:10.1007/978-3-319-24574-4_28.

12. Schindelin J, Arganda-Carreras I, Frise E. et al. Fiji: an open-source platform for biological-image analysis. Nat Methods. 2012; doi:10.1038/nmeth.2019.

Figure 1

Click here to access/download;Figure;fig1.tif

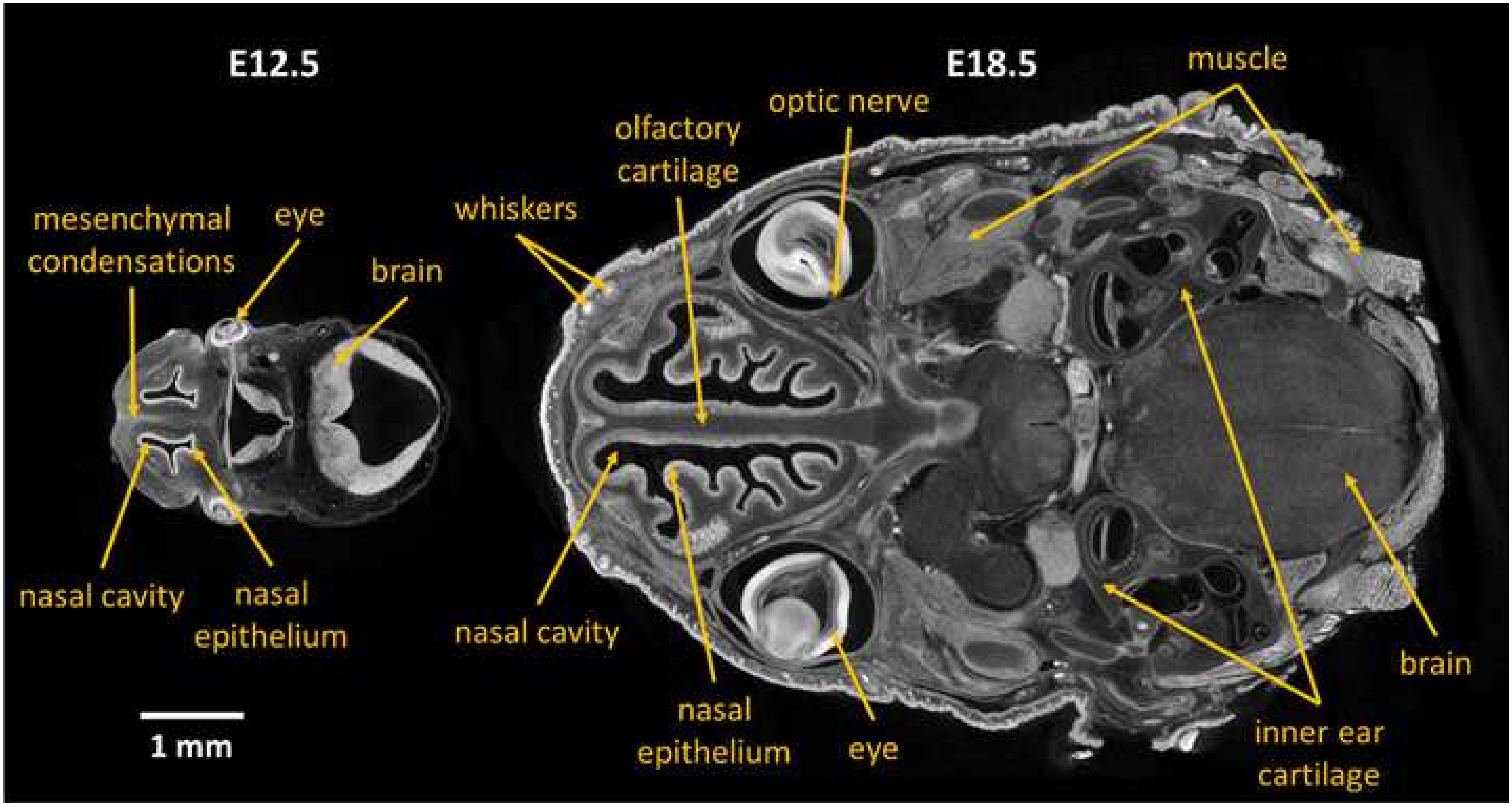

Figure 2

Click here to access/download;Figure;fig2.tif

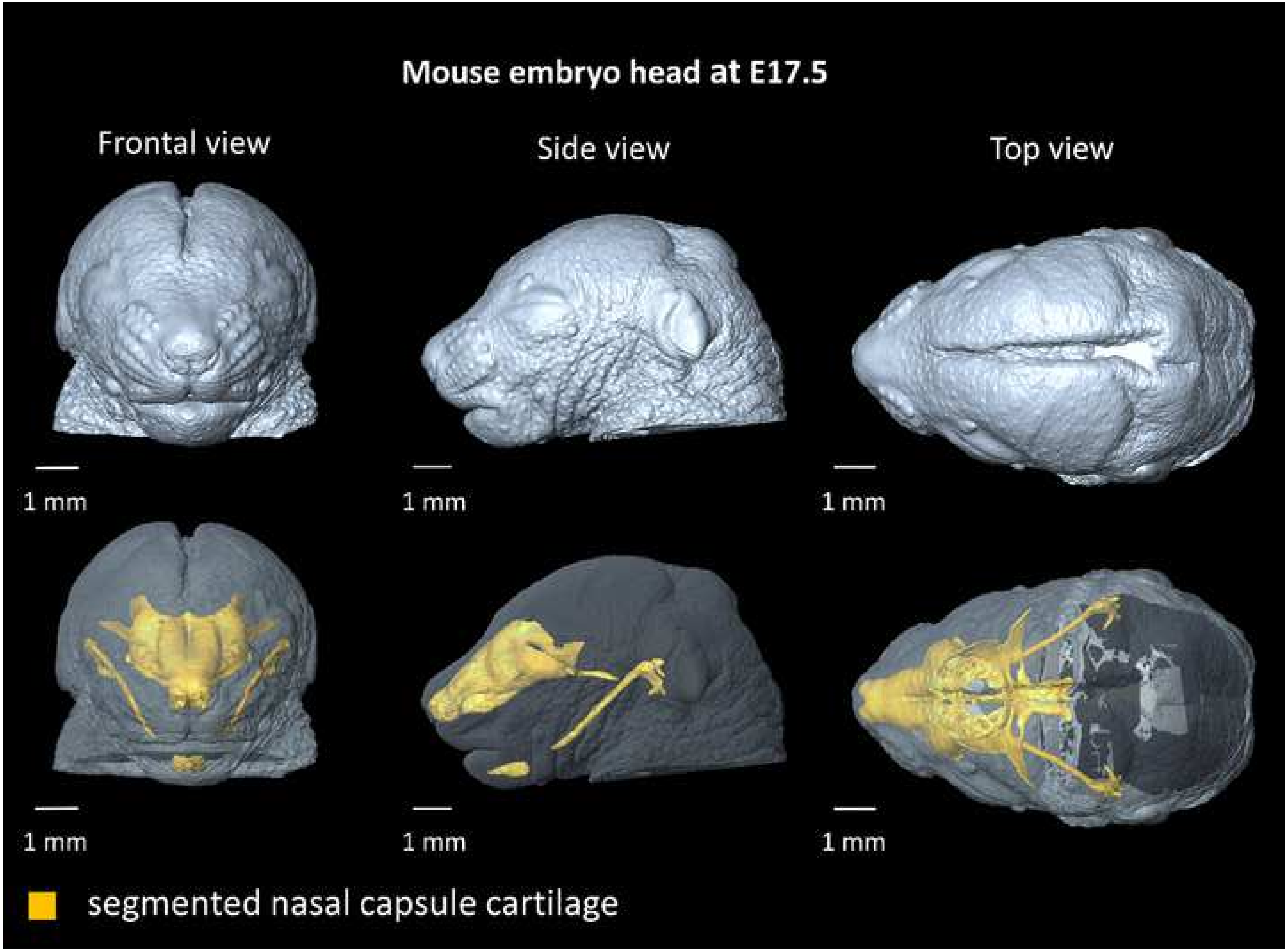

Figure 3

Click here to access/download;Figure;fig3.tif

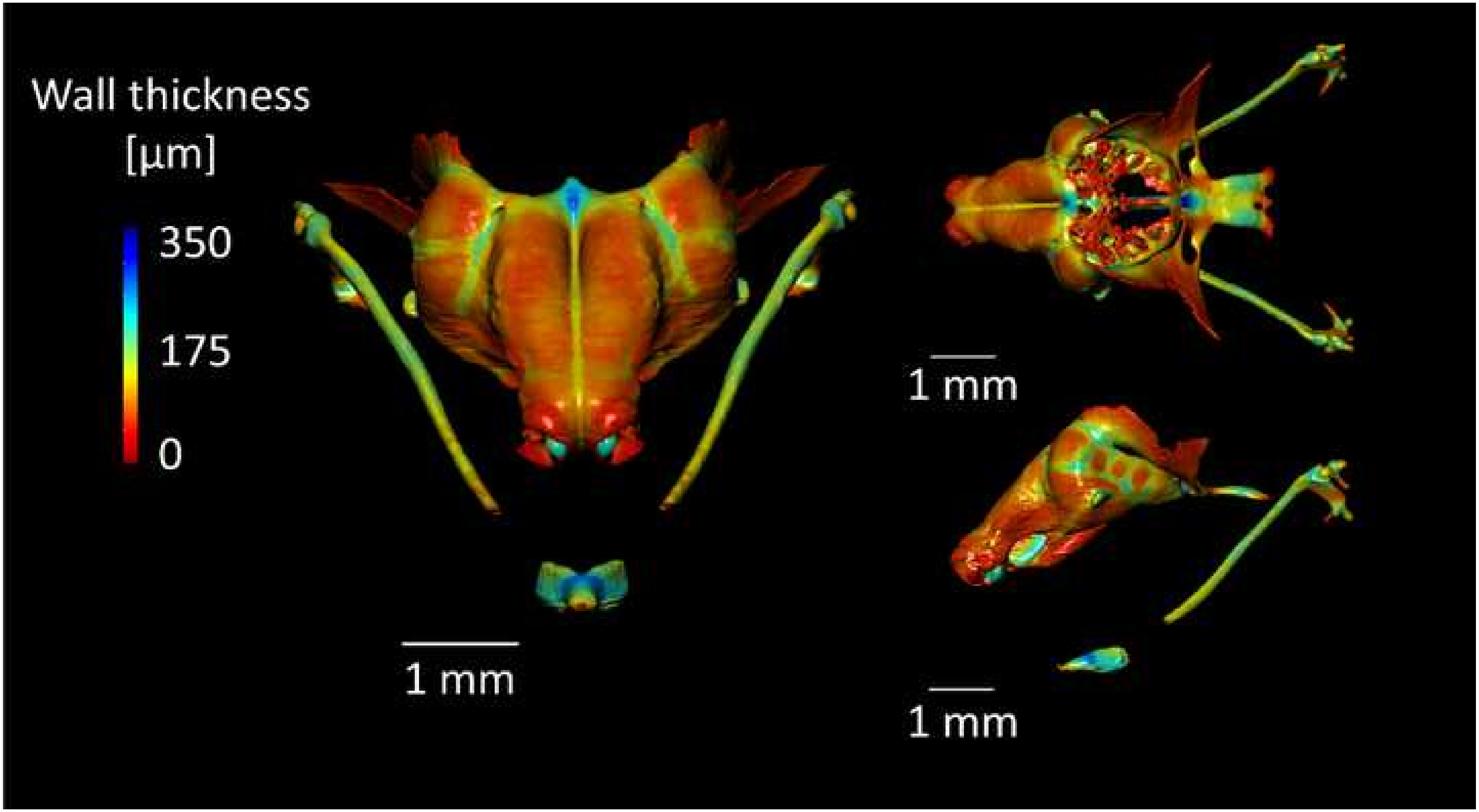

Figure 4

Click here to access/download;Figure;fig4.tif

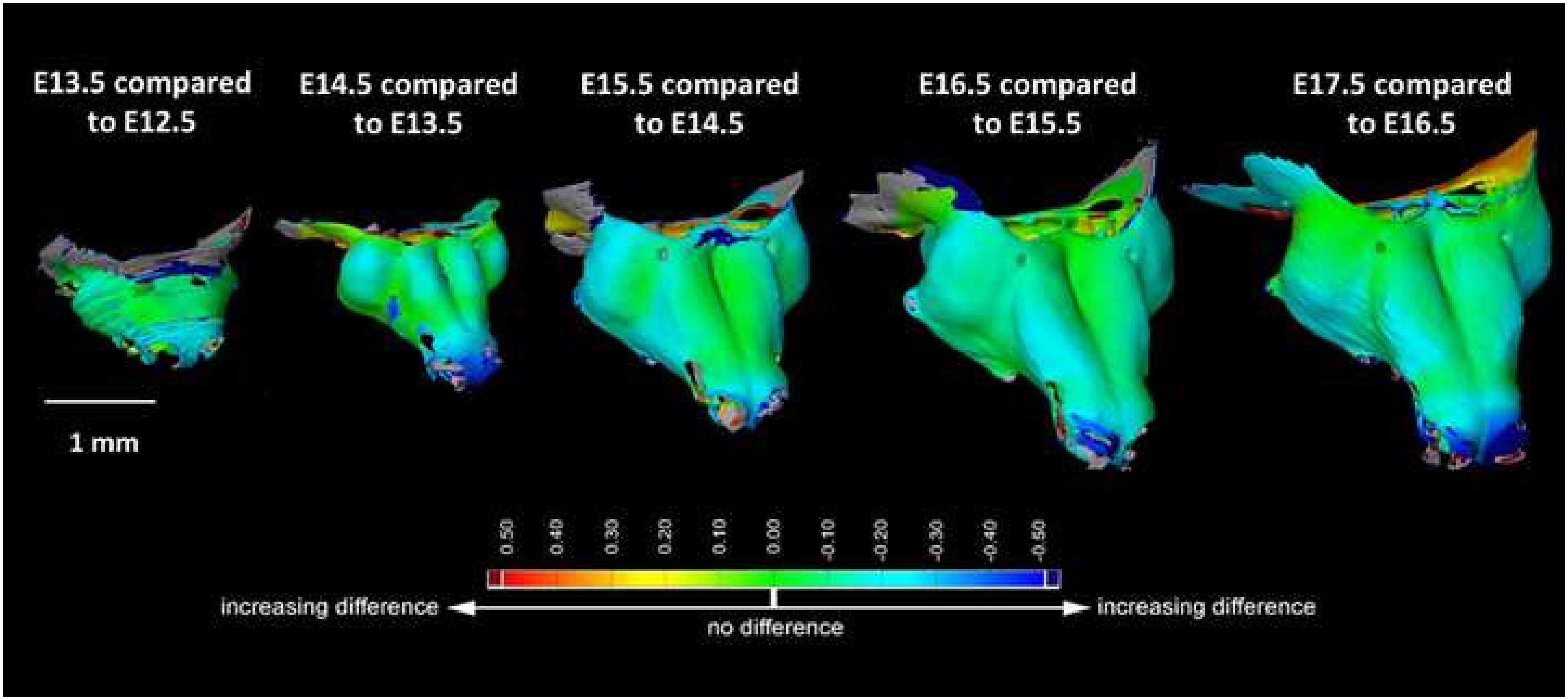

Figure 5

Click here to access/download;Figure;fig5.tif

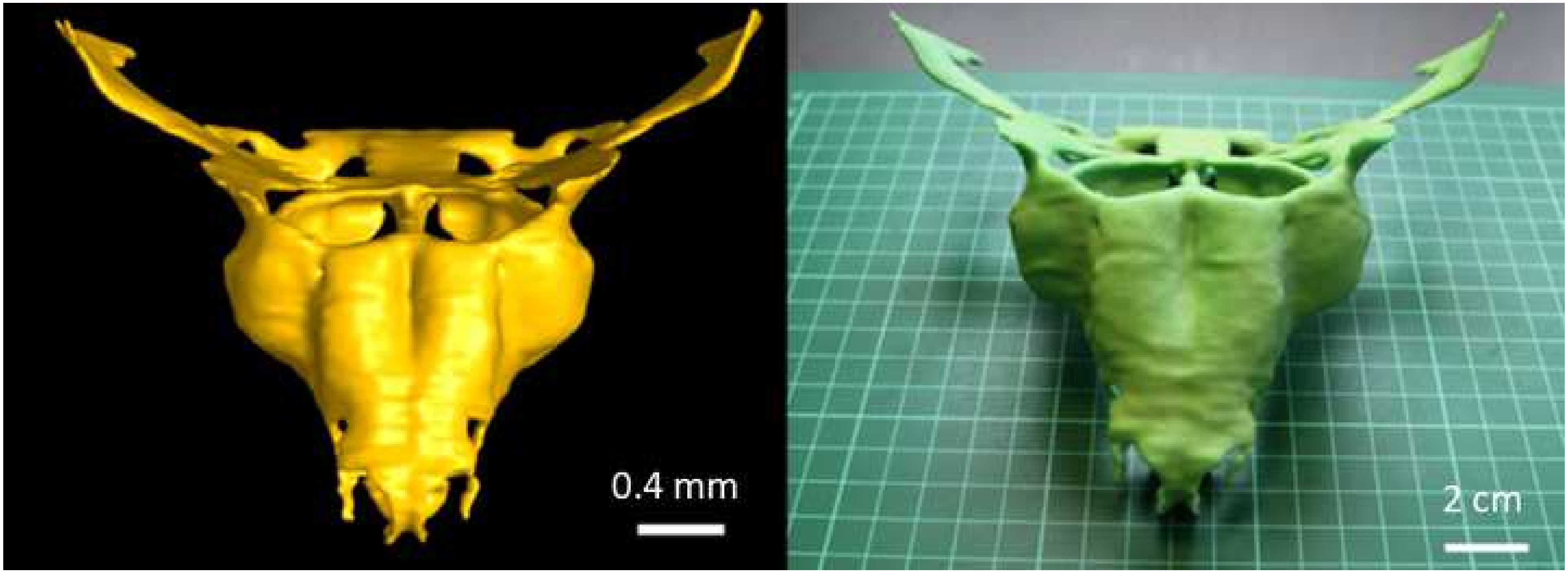

Supplement: giab012_GIGA-D-20-00282_Original_Submission [file giab012_giga-d-20-00282_original_submission.pdf]
