# Supplementary material for: X-ray microtomography–based atlas of mouse cranial development
Source: Gigascience. 2021 Mar 2;10(3):giab012. doi: 10.1093/gigascience/giab012 (PMC7936920; doi:10.1093/gigascience/giab012)

GigaScience

X-ray microtomography-based atlas of mouse cranial development

--Manuscript Draft--

**Manuscript Number:**	GIGA-D-20-00282R1
**Full Title:**	X-ray microtomography-based atlas of mouse cranial development
**Article Type:**	Data Note
**Funding Information:**	Ministerstvo Školství, Mládeže a Tělovýchovy (LQ1601/LM2018110) — Not applicable<br>Vysoké Učení Technické v Brně (CEITEC VUT-J-20-6477) — Mr. Jan Matula<br>Vysoké Učení Technické v Brně (CEITEC VUT-J-20-6317) — Mrs. Marketa Tesarova

Abstract:

Background

X-ray microtomography (μCT) has become an invaluable tool for non-destructive analysis of biological samples in the field of developmental biology. Mouse embryos are a typical model for investigation of human developmental diseases. By obtaining 3-D high resolution scans of the mouse embryo heads, we gain valuable morphological information about the structures prominent in the development of future face, brain and sensory organs. The development of facial skeleton tracked in these μCT data provides a valuable background for further studies of congenital craniofacial pathologies and normal development.

Findings

In this work, re-usable tomographic data from 7 full 3-D scans of mouse embryo heads are presented and made publicly available. The ages of these embryos range from E12.5 to E18.5. The samples were stained by phosphotungstic acid prior to scanning, which greatly enhanced the contrast of various tissues in the reconstructed images and enabled precise segmentation. The images were obtained on a lab-based μCT system. Furthermore, we provide manually segmented masks of mesenchymal condensations (for E12.5 and E13.5) and cartilage present in the nasal capsule of the scanned embryos.

Conclusion

We present a comprehensive dataset of X-ray 3-D computed tomography images of the developing mouse head with high-quality manual segmentation masks of cartilaginous nasal capsules. The provided μCT images can be used for studying any other major structure within the developing mouse heads. The high quality of the manually segmented models of nasal capsules may be instrumental to understand the complex process of the development of the face in mouse model.

**Corresponding Author:**	Tomas Zikmund, PhD<br>Central European Institute of Technology, Brno University of Technology<br>Brno, CZECH REPUBLIC
**Corresponding Author Secondary Information:**	
**Corresponding Author's Institution:**	Central European Institute of Technology, Brno University of Technology
**Corresponding Author's Secondary Institution:**	
**First Author:**	Jan Matula, MSc
**First Author Secondary Information:**	
**Order of Authors:**	Jan Matula, MSc
	Marketa Tesarova, MSc
	Tomas Zikmund, PhD

Powered by Editorial Manager® and ProduXion Manager® from Aries Systems Corporation

	Marketa Kaucka, PhD
	Igor Adameyko, PhD
	Jozef Kaiser, PhD
**Order of Authors Secondary Information:**	
**Response to Reviewers:**	Reviewer #1: The authors present a set of 3D images of mouse embryo heads from daily stages particularly important for facial skeletal development (E12-18). These are high-quality and complete contrast-enhanced micro-CT images showing details of soft tissues at the level of low-magnification histology. They will be potentially useful in any number of embryological studies. The dataset also includes segmentations (delimited sub-volumes, or masks) of the developing facial cartilage, from another publication. This is a model submission for presenting a dataset of this kind.<br>In the Word document, I have offered some suggestions for slight improvements to the style and presentation. Otherwise, the report appears ready for publication.<br><br>Response: We are grateful to the reviewer for their kind words. We appreciate the suggestions in the attached word document, and we incorporated them into the manuscript where we found it appropriate.<br><br>Reviewer #2: The manuscript by Matula et al demonstrated the use of novel micro-CT imaging to visualize mouse embryo cranial development. The manuscript is well-written overall with high-quality micro-CT images. However, the authors only used one embryo in each of timepoints.<br>1.In sample preparation, authors mention that the embryos undergo dehydration and rehydration. These processes cause significant shrinkage and expansion of the sample. Hence, the results may present distorted structural features.<br><br>Response: We are thankful to the reviewer for this comment. The 3-D segmentation of nasal capsule and Meckel cartilage in presented in this work corresponds with visualizations of this structure by other techniques (PMID: 28414273). We are aware that shrinkage may occur, and for this reason, the dehydration and rehydration are performed as slow as possible (30%, 50% and 70% methanol for 1 day each) to minimize shrinking of the embryonic tissue. Dehydration and rehydration are a standard process when staining by PTA and it has been used for wide application in developmental studies (10.1002/dvdy.136).<br><br>Reviewer #2: Why are voxel size differ between samples? This is problematic when comparing different samples. Especially for future studies comparing morphological features between samples (i.e. different age, treatment, mouse genetic studies).<br><br>Response: The general strategy is to always obtain data of the highest possible resolution for each sample. In order to achieve the best possible resolution for each sample, the scanning parameters are modified for each sample separately because of the individual sample size. This is driven by the geometric magnification of the CT system; state-of-the-art systems provide voxel size of approximately 1/1000 of the sample size.<br>As almost every software designed to work with 3-D imaging data respects the given voxel size, morphological comparison between the group of scans or even scans created with completely different voxel sizes, should pose no issues. If it is necessary to have scans of the same voxel size, it is possible to resample the provided data to a unified voxel size, and still keep the resolution high, but this would degrade the quality of the image data with the highest resolution and this is not something we feel would benefit the dataset.<br><br>Reviewer #2: The study reused existing micro-CT scans to analyse mouse cranial development. The image quality is high but only one embryo was scanned in each time point. The study would be significantly improved if authors can demonstrate its variability or reproducibility by scanning multiple embryos (>3 embryos/timepoint).<br><br>Response: The goal of this published set of data is not to show variability among developmental groups, but to provide a compact, easy-to-work-with dataset, that can be quickly downloaded and processed and represents the general development of the mouse embryo in the set time range. This is further highlighted by the Theiler staging

Powered by Editorial Manager® and ProduXion Manager® from Aries Systems Corporation

process performed per the suggestion of Reviewer #3. Furthermore, we do not try to quantify any variability in this manuscript since it is highly dependent on the specific biological application.

Reviewer #2: Can authors show any quantification? For example, measuring the length of nasal capsule cartilage or volume of nasal cavity. The quantification is possible with micro-CT and AVIZO software

Response: We agree with the reviewer, that some general quantification of the morphology would benefit the data validation and quality control section of the manuscript. We included measurements of nasal capsule morphology in terms of its length and width (lines 154, 161-162, table 3). This also shows a general progress in the growth of the nasal capsule and introduce a potential reader to the dimensions present. More specific quantification would be again highly dependent on the particular application utilizing the data can be selected.

Reviewer #2: The study used phosphotungstic acid (PTA) as a contrast staining. Have they tested other staining methods such as Lugol's Iodine and Osmium Tetraoxide staining? These staining methods are more widely used(e.g. PMID: 27513872, 27345427, 28892037).

Response: The focus of the study utilizing this dataset is mainly the investigation of craniofacial development. PTA offers unparalleled contrast in imaging of cartilaginous tissues in comparison to the other suggested staining solutions (as the contrasting solution does not penetrate the cartilaginous tissues and as a result, visible difference can be observed in attenuation of cartilage and the surrounding tissues. The trade-off is the long staining time as it takes time for the large molecule of PTA to fully penetrate the sample. We may have neglected to mention the other, more widely utilized staining methods, which is why we added a note on these with relevant literature to the Context section of the manuscript (lines 73-74).

Reviewer #2: 1% agarose gel. Need to specify which type of agarose was used. Low temperature agarose gel should have been used to avoid any sample damage as regular agarose gel has high melting point which can destruct the sample

Response: We are grateful to the reviewer for this comment. During the revision of the manuscript, we found an error in the concentration of the utilized agarose. We deeply apologize for this mistake and corrected the value (1% agarose to 0.5% agarose). The agarose utilized in this work is A5304, Sigma-Aldrich. This agarose is not of the low-melting point type, but special care is given to make sure no damage occurs to the sample during its fixation. After its dissolution in boiling water, the agarose is left to cool to safe temperature (45 °C), while it still remains fluid and only then it is used for the sample fixation. The information about the agarose type was included to the manuscript (line 104).

Reviewer #2: There is huge difference in sample incubation in PTA solution. E12.5 was stained for 7 days, E15.5 for 21 days, E18.5 for 49 days. Can authors optimise the incubation procedure to reduce incubation time? Perhaps using different contrast staining methods (i.e. Lugol's Iodine or Osmium Tetraoxide)

Response: It is true the staining times are very high in comparison to other staining methods. The answer to the reviewer's question about the long staining time was partially answered in response to the minor issue #1. The staining used for the presented samples was focused on making the nasal capsule as distinguishable as possible for the operator to be able to perform the manual segmentation of the mesenchymal condensations/cartilage. We also experimented with the contrast staining methods the reviewer mentions, but we achieved the greatest contrast with PTA (phosphotungstic acid) with the obvious trade-off of much longer staining times, to allow the contrast to fully penetrate the tissues due to its large molecule size.

Reviewer #2: How were the micro-CT scanning parameters determined for optimal quality? Also, how long was each scan?

Response: The scanning time with the 2000 projections and 900 ms exposure time

with triple averaging of the X-ray projections was about 1 h 30 min. The information about the scanning time was included to the manuscript (lines 120-121). These scanning parameters are a result of optimization to achieve the best possible image quality with a reasonable scanning time. We experimented with several setups of X-ray tube (accelerating voltage, current and filtration) and detector (averaging, number of projections, exposure time). The resulting data quality were evaluated terms of contrast, which allowed precise segmentation, and the signal-to-noise ratio.

Reviewer #2: Line 130-132 “This three-fold increase in segmentation speed does not affect the accuracy of the segmentation in a significant way...”. Needs referencing or data to support the statement.

Response: To support this statement, we performed an experiment with a manually segmented cartilaginous nasal capsule, that was segmented on a slice-by-slice basis. From this data, we selected only every 3rd slice in the plane, where the segmentation was performed and then utilized the same interpolation process as was used in this work. The result of this experiment is that there is a 98% overlap (Dice coefficient) between the volume utilizing interslice interpolation and dataset utilizing slice-by-slice segmentation in the case of this type of sample. This information was added to the manuscript (lines 138-140).

Reviewer #3: This Data Note describes a microCT dataset of mouse embryonic development. The image data is of high quality and high contrast, and consists of 8-bit TIFF stacks with an impressive 3-micron isotropic voxel resolution for the E13.5 specimen. The authors are to be commended for using the contrast agent phosphotungstic acid to improve the quality of the microCT images. In addition, the authors provide manually segmented masks of mesenchymal condensations and nasal capsule cartilage, and the authors claim that these can be used to measure and understand morphological features of cranial development, such as chondrocranium fusion events. The authors further highlight the reuse potential of the manually segmented masks for developing machine-learning approaches for automated segmentation. I think that this is an interesting use case for these data, and I commend the authors for making these data publicly available in the GigaScience DataBase.
Major comment 1 The authors highlight the reuse potential of the microCT data for exploring developmental changes in craniofacial morphology between E12.5 and E18.5. On this note, there are existing histological atlases detailing craniofacial development, such as Kaufman's 'The Atlas of Mouse Development', that use Theiler staging, which is a morphological staging system developed by Karl Theiler to accurately stage mouse embryos. The reason why a morphological staging system is important is because in a litter of mouse embryos of the same age, some embryos are observably more advanced in development than others. One method of handling this inherent biological variation that one observes in age-matched littermates is to further stage the embryos based on morphological criteria. With this in mind, I invite the authors to Theiler stage each of the seven mouse embryo models in this dataset. This would be a great asset and would allow a researcher to compare the microCT data outlined in this Data Note to stage-matched anatomical atlas models, such as those used in the Kaufman Atlas and the eHistology resource (https://www.emouseatlas.org/emap/eHistology/). Of note, online resources are available to assist in the Theiler staging process:
https://www.emouseatlas.org/emap/ema/theiler_stages/StageDefinition/stagedefinition.html In addition, Karl Theiler's book on Theiler staging is openly available at the following link:
https://www.emouseatlas.org/emap/ema/theiler_stages/house_mouse/book.html

Response: We would like to thank the reviewer for this very helpful suggestion and for providing the links to relevant resources. We see how important this type of staging is for further re-use of this data. For this reason, we performed Theiler staging and incorporated the information about the embryos Theiler stages into the manuscript (Table 1, column Theiler stage, lines 107-110).

Reviewer #3: Whereas I can open the TIFF stacks of the microCT image data in Fiji / ImageJ and I can observe grayscale image data, the corresponding mask image data just appears as a black image. Can the authors provide instructions on how to view the manually segmented masks using Fiji / ImageJ? The example dataset I am using for

	Fiji / ImageJ is the E13.5 dataset.<br><br>Response: As the masks are saved as 8-bit tiff image and contains only values 0 (background) and 1 (mesenchymal condensation/cartilage), it will be, by many image viewers, displayed as almost black image, because they are using the whole range from 0 to 255 to display the image. To view the segmentation, it is necessary to set the display window in the selected software to the range 0 to 1. The segmentation will then be displayed as white on black background. We see how this could be confusing for potential users of the data. We modified the data re-use section of the manuscript with this information (219-222).<br><br>Reviewer #3: Figure 2 shows a 3D surface reconstruction of an E17.5 mouse embryo head. I see great value in surface reconstructions of the 3D models as they allow researchers to explore the external morphology of each of the specimens. Can the authors please clarify whether they will be submitting 3D surface reconstructions (e.g. STL format) of all seven models to the GigaScience DataBase?<br><br>Response: We agree with the reviewer, that seeing the 3-D rendering of the embryos will help potential re-users of this dataset. We therefore include 3-D .stl files alongside the data showing the embryo heads. Appropriate text was added to the data availability section of the manuscript on how to work with these files (lines 210-211, 222-223).<br><br>In addition to the reviewers comments, two errors in the manuscript were found during the revision process. Firstly, in the rehydratation part of the staining protocol, ethanol was mistakenly stated instead of methanol. This error in the manuscript was corrected on line 103. Additionally, when stating voxel size of the Embryo 1 sample, by error we used a value of 3.2 μm. We corrected this value to the correct value of 2.6 μm in the Table 2 of the manuscript. This error was also corrected in the submitted data. We deeply apologize for these mistakes. Furthermore, we would like to express our gratitude to the reviewers for their helpful comments.
**Additional Information:**	
**Question**	**Response**
Are you submitting this manuscript to a special series or article collection?	No
**Experimental design and statistics**<br><br>Full details of the experimental design and statistical methods used should be given in the Methods section, as detailed in our Minimum Standards Reporting Checklist. Information essential to interpreting the data presented should be made available in the figure legends.<br><br>Have you included all the information requested in your manuscript?	Yes
**Resources**<br><br>A description of all resources used, including antibodies, cell lines, animals and software tools, with enough information to allow them to be uniquely	Yes

identified, should be included in the Methods section. Authors are strongly encouraged to cite Research Resource Identifiers (RRIDs) for antibodies, model organisms and tools, where possible.<br><br>Have you included the information requested as detailed in our Minimum Standards Reporting Checklist?	
**Availability of data and materials**<br><br>All datasets and code on which the conclusions of the paper rely must be either included in your submission or deposited in publicly available repositories (where available and ethically appropriate), referencing such data using a unique identifier in the references and in the “Availability of Data and Materials” section of your manuscript.<br><br>Have you have met the above requirement as detailed in our Minimum Standards Reporting Checklist?	Yes

Manuscript
Click here to access/download;Manuscript;data_note_reviewed_fin_SCE.do

X-ray microtomography-based atlas of mouse cranial development

Jan Matula[1], Marketa Tesarova[1], Tomas Zikmund[1], Marketa Kaucka[2,3], Igor Adameyko[3], Jozef Kaiser[1]

[1] Central European Institute of Technology, Brno University of Technology, Brno, Czech Republic

[2] Max Planck Institute for Evolutionary Biology, Plön, Germany

[3] Medical University Vienna, Vienna, Austria

ORCID IDs: Jan Matula: 0000-0003-3334-956X; Marketa Tesarova: 0000-0002-5200-7365; Tomas Zikmund: 0000-0003-2948-5198; Marketa Kaucka: 0000-0002-8781-9769; Igor Adameyko: 0000-0001-5471-0356; Josef Kaiser:

Abstract

Background: X-ray microtomography (µCT) has become an invaluable tool for non-destructive analysis of biological samples in the field of developmental biology. Mouse embryos are a typical model for investigation of human developmental diseases. By obtaining 3-D high resolution scans of the mouse embryo heads, we gain valuable morphological information about the structures prominent in the development of future face, brain and sensory organs. The development of facial skeleton tracked in these µCT data provides a valuable background for further studies of congenital craniofacial pathologies and normal development.

Findings: In this work, re-usable tomographic data from 7 full 3-D scans of mouse embryo heads are presented and made publicly available. The ages of these embryos range from E12.5 to E18.5. The samples were stained by phosphotungstic acid prior to scanning, which greatly enhanced the contrast of various tissues in the reconstructed images and enabled precise segmentation. The images were obtained on a lab-based µCT system. Furthermore, we provide manually segmented masks of

mesenchymal condensations (for E12.5 and E13.5) and cartilage present in the nasal capsule of the scanned embryos.

Conclusion: We present a comprehensive dataset of X-ray 3-D computed tomography images of the developing mouse head with high-quality manual segmentation masks of cartilaginous nasal capsules. The provided µCT images can be used for studying any other major structure within the developing mouse heads. The high quality of the manually segmented models of nasal capsules may be instrumental to understand the complex process of the development of the face in mouse model.

Keywords

X-ray, computed tomography, microtomography, mouse embryo head, tissue contrast, 3-D modelling, nasal capsule

Data description

Context

The vertebrate head is considered one of the most complex parts of the body. The head is formed during the embryonic development through a process known as morphogenesis, which involves hundreds of genes and non-coding regulatory sequences [1,2]. This intricate body compartment hosts numerous cell and tissue types forming, for instance, muscles, ligaments, nerves and central nervous system, sensory organs, hair follicles, teeth, which are all integrated in the complexly shaped skull. There is a remarkable inter- but in some cases (such as humans) also intra-species variability of the craniofacial shapes [3]. Reportedly, the shape of the face (or the whole head) depends on the geometry of the skeleton that provides protection to sensitive nervous tissues and serves as a scaffold for muscle attachment [1]. The skeleton of the head is formed by two types of stiff tissue – bone and cartilage. Although the majority of the head skeleton in mammals is formed by bones postnatally, the embryonic development of the skull relies on the cartilage. Chondrocranium is induced as 14 independent pieces that grow, acquire specific shape and fuse later to form the skull [1]. Interestingly,

the development of cartilage and bone corresponds to the progress of development of central and peripheral nervous system and sensory organs [2]. Therefore, the exact developmental link between the emergence of nervous structures and the appearance of cartilage and bone is one of the fundamental questions in developmental biology. At the same time, understanding both the molecular basis and cellular dynamics driving the formation and shaping of the mammalian head is of utmost interest in the fields of clinical genetics and regenerative medicine, dealing with a broad spectrum of human congenital craniofacial disorders.

In our previous work, we aimed to explore the exact sequence of formation and shaping of the developing mammalian face and we used a mouse model for our investigation [1,2]. The morphological properties of the observed structures are complex, and to fully understand their shaping, advanced imaging techniques are required. X-ray computed tomography technique is one of the oldest imaging techniques, but in recent years it has shown its strengths in the field of developmental biology [4]. The principle of X-ray computed tomography lies at acquiring 2-D projections of the scanned sample at regular angle increments. A 3-D view of the scene is then created by the process of tomographic reconstruction. This way we gain 3-D spatial information that would be otherwise unobtainable without destroying the sample. The superior resolution of modern lab-based µCT machines provides a way to visualize and analyze biological structures on the level of micrometres and, more importantly, in the 3-D spatial context. We combined genetic tracing, gene knock-out strategies, mathematical modelling and µCT to reconstruct the craniofacial development in detail. As a result, we generated a set of µCT scans from wild type mouse strains, ranging from E12.5 (where the first induction of early cartilage, represented by condensation of the mesenchyme, can be observed) to E18.5 with fully formed chondrocranium.

While µCT has been proven useful for non-destructive high-resolution imaging of high-density biological tissues (e.g. bones [5,6], teeth [7,8]), there are issues with the differentiation between types of soft tissues in the resulting images. The reason is an insufficient difference in their X-ray attenuation

coefficients, which results in low contrast in the reconstructed tomographic images [4]. This inherent limitation of absorption-based computed tomography can be addressed by utilizing contrast-enhancing techniques (e.g. staining the sample with contrast-enhancing chemical substances [9]). Several approaches for soft tissue contrasting are explored in literature, e. g. Osmium tetroxide [10,11], Lugol's iodine [12,13] or phosphotungstic acid (PTA) [4,9]. We used a tissue-contrasting method based on a different uptake of PTA by various tissues, resulting in excellent resolution and visibility of fine structures (see Figure 1 for example tomographic slices). It enabled us to differentiate between nasal capsule cartilage (and mesenchymal condensations in the images of younger embryos) and surrounding soft tissues. An operator was then able to manually segment the mesenchymal condensations and cartilage forming the nasal capsule of the embryos (Figure 2). We provide the generated manual segmentations alongside the tomographic slices. These scans can be used by researchers interested in the development of various structures in the head.

Figure 1: Examples of tomographic slices of mouse embryos 12.5 days old (E12.5) and 18.5 days old (E18.5). µCT scanning of samples stained with PTA provides image data with excellent contrast, where even fine details are visible. Yellow arrows show areas of the imaged that might be interesting for potential users of the provided dataset.

Figure 2: 3-D reconstruction of a mouse embryo head E17.5. Yellow 3-D model represents segmented nasal capsule and Meckel cartilage in the head.

The provided atlas of mouse cranial development (including tomographic slices and segmented nasal capsules) will be essential for tracing normal development of any tissue type within the vertebrate head. Given the excellent differential contrast and general high quality of the data, they can be re-used for any investigation of normal anatomy during developmental time course.

Methods

Sample preparation

Mouse embryonic heads were contrasted using PTA-staining procedure, followed by a µCT measurement. The staining protocol is a modification of the protocol pioneered by Brian Metscher [9]

and has been described previously in [2,14]. Briefly, the mouse embryos were fixed with 4% formaldehyde in phosphate buffer saline (PBS) for 24 hours at 4 °C. The samples were then washed with PBS and subsequently dehydrated with 30%, 50% and 70% ethanol for 1 day each to minimize shrinking of the embryonic tissue. The samples were then transferred into 0.5-1.0% PTA solution (Lach-Ner, Czech Republic) in 90% methanol. The solution was replaced every 2 to 3 days. The E12.5 sample was left to absorb the contrasting solution for 1 week, the E15.5 for 3 weeks and the E18.5 for 7 weeks. After the stage E15.5, the head was separated from the body at the level of shoulders to ensure an adequate and uniform contrasting. After this staining procedure was completed, the samples were rehydrated in methanol series of decreasing concentration (90 %, 70 %, 50 % and 30 %). Prior to the µCT scanning, the samples were submerged in 0.5% agarose gel (A5304, Sigma-Aldrich) and placed in polypropylene conical tubes with volume ranging from 0.5 to 15 ml. The tube volume was chosen with respect to the size of the sample in order obtain images of the best possible quality. To further characterize the embryonal stages in addition to their age after fertilization, Theiler staging was performed [15]. The prepared samples, together with additional information, are listed in Table 1.

Table 1: List of samples

Resource	Organism	Strain	Age after fertilization	Theiler stage	Source	RRID
Embryo 1	*Mus musculus*	C57BL/6NCrl	12.5 days	TS 20	Charles River Germany	RRID:IMSR_CRL:27
Embryo 2	*Mus musculus*	C57BL/6NCrl	13.5 days	TS 21	Charles River Germany	RRID:IMSR_CRL:27
Embryo 3	*Mus musculus*	C57BL/6NCrl	14.5 days	TS 23	Charles River Germany	RRID:IMSR_CRL:27
Embryo 4	*Mus musculus*	C57BL/6NCrl	15.5 days	TS 24	Charles River Germany	RRID:IMSR_CRL:27
Embryo 5	*Mus musculus*	C57BL/6NCrl	16.5 days	TS 25	Charles River Germany	RRID:IMSR_CRL:27
Embryo 6	*Mus musculus*	C57BL/6NCrl	17.5 days	TS 26	Charles River Germany	RRID:IMSR_CRL:27

Embryo 7	*Mus musculus*	C57BL/6NCrl	18.5 days	TS 26	Charles River Germany	RRID:IMSR_CRL:27

Image acquisition

The samples were scanned with a lab-based µCT system GE Phoenix v|tome|x L 240 (GE Sensing & Inspection Technologies GmbH Germany). The system was equipped with a high contrast flat panel detector DXR250 with 2048 × 2048 pixel resolution. The embryos were fixed in polypropylene conical tubes with 0.5% agarose gel to prevent the sample movement during the µCT stage rotation. 2000 projections were acquired with an exposure time of 900 ms per projection. Each projection was captured three times and an average of the signal was used to improve the signal-to-noise ratio. The acceleration voltage of the X-ray tube was 60 kV and the tube current 200 µA. The X-ray beam was filtered with a 0.1 mm aluminium plate. The time required for scanning one sample was 1 hour and 30 minutes.

Software processing

Tomographic reconstruction of the obtained set of projections was performed with GE phoenix datos |x 2.0 3-D computed tomography software (GE Sensing & Inspection Technologies GmbH Germany), which allowed to generate a 3-D image of the mouse embryo head. The voxels are isotropic, the voxel sizes for individual samples are shown in Table 2.

Table 2: Voxel sizes of individual samples

Sample	Voxel size [µm]
Embryo 1	2.6
Embryo 2	3
Embryo 3	5
Embryo 4	6
Embryo 5	6

Embryo 6	5.8
Embryo 7	5.5

Manual segmentation

Avizo (Thermo Fisher Scientific, USA) image processing software was used for manual segmentation of the mesenchymal condensations and nasal capsule cartilage in the reconstructed CT images. Avizo is a commercial software providing a broad range of tools for manipulating and procession 3-D image data. The manual segmentation of the cartilaginous nasal capsule tissue takes approximately 10 to 20 hours depending on the size of the sample and the experience of the operator. To make the load of 3-D segmentation volume smaller, only every 3rd slice was manually segmented and the rest was calculated by linear interpolation between adjacent manually segmented slices [14]. This three-fold increase in segmentation speed does not affect the accuracy of the segmentation in a significant way, because the small slice width makes differences in structures in adjacent slices minimal. The overlap between the segmentation performed on a slice-by-slice basis and segmentation with the interslice interpolation is over 98 % (Dice coefficient) in the case of this type of sample. The cartilage was segmented in 2-D slices of the whole 3-D volume, so there is in some cases a staircase artefact present in the planes other than the plane in which the segmentation was performed.

Data validation and quality control

The segmented 3-D models of nasal capsule can be subjected to various subsequent analyses that further highlight the differences between compared models from distinct samples. For instance, wall thickness analysis of the segmented nasal capsule provides valuable information outside of the general morphology assessment of the mouse embryonic anterior face. This information serves to compare multiple samples and provides quantitative information on the variability within each specimen (Figure 3). Such an approach was instrumental in the work of Kaucka and collaborators [1,2] where the wall thickness analysis was used to dissect the fundamental mechanisms of cartilage growth

and highlighted the molecular basis of the thickness regulation. The obtained results were implemented in a mathematical model that could predict the underlying cellular dynamics of the cartilage growth. Furthermore, using this method it was possible to depict subtle differences between control and mutant embryonic samples that appeared otherwise morphologically similar [1]. Together with core measurements such as the width and the length (see Table 3) of the nasal capsule and mapping the surface expansion during the embryonic growth, authors acquired detailed understanding of the shaping and the growth of this complex structure.

Figure 3: Wall thickness analysis of the manually segmented mouse embryonic nasal capsule (sample E17.5). The wall thickness is calculated as the diameter of a hypothetical sphere that fits within boundary points of the nasal capsule mesh. The 3-D wall thickness model was created in the Dragonfly software (Object Research Systems (ORS) Inc., Canada).

Table 3: Length and width measurement of the manually segmented nasal capsule performed in the Avizo (Thermo Fisher Scientific, USA) software with the Measure tool

Sample	Length [mm]	Width [mm]
Embryo 1	0.48	1.37
Embryo 2	0.90	1.41
Embryo 3	1.33	1.53
Embryo 4	1.56	1.99
Embryo 5	2.12	2.53
Embryo 6	2.34	2.83
Embryo 7	2.56	2.85

Shape comparison between individual stages of development provides us with valuable information about the areas of the sample, where growth is the most prominent. Figure 4 depicts such analysis performed on nasal capsule of embryos in developmental stages ranging from 12.5 to 17.5 days old [1]. This analysis was done in the software GOM Inspect (GOM GmbH, Germany).

Figure 4: Manually segmented nasal capsules of developmental stages E13.5, E14.5, E15.5, E16.5 and E17.5 were compared to the previous developmental stage in the GOM Inspect Software. Figure adapted from Figure 3—figure supplement 2 from [1] *under CC BY 4.0.*

By manually segmenting the nasal capsule cartilage in reconstructed images of the samples, we were able to obtain anatomically accurate 3-D printed model of the embryonic mouse nasal capsule. This is very beneficial for researchers to physically evaluate the morphology of the embryonic head. Precise visualisation of the developing nasal capsule together with the opportunity to produce a physical 3-D-printed model of this complex anatomical structure allows better understanding of the organization of single skeletal elements in the framework of the sophisticated organisation of mammalian embryonic head [14]. (Figure 5).

Figure 5: 3-D printed model of the mouse embryo nasal capsule (right) next to its 3-D render created from manually segmented binary masks (left). Figure adapted from Figure 7 from [14] *under CC BY 3.0.*

Re-use potential

This dataset with its high quality manually segmented masks can be instrumental in creating a robust method for segmentation of cartilaginous structures from µCT images of mouse embryos. The field of image processing is lately being dominated by deep learning algorithms and specifically convolutional neural networks (CNNs) consistently achieve state-of-the-art results in fully-automatic image segmentation tasks [16]. A segmentation model created in such way could make acquiring new samples for analysis of nasal capsule development in mouse embryos much less time consuming, because the time-expensive process of manual segmentation would be eliminated. Nevertheless, high quality scans with a sufficient tissue contrast are required for such automated segmentation. Our dataset has been validated for its suitability for such deep learning algorithm application and can be therefore used by other researchers for this purpose as well.

Biological potential:

The possibilities to re-use this dataset are broad and include the analysis of developmental changes in nasal epithelium, eyes, whiskers, tongue, oral cavity, developing teeth, brain, cranial cartilage and bone, tendons, muscles, endocrine organs, vessels and nerves. For instance, questions pertaining to the mechanisms controlling growth and shaping of the brain or craniofacial skeleton are still open and will benefit from presented data. Furthermore, during development and growth, multiple tissue-interactions and integration events occur at multiple morphologically distinct tissue interfaces. Such interactions at the tissue scale lead to the development of muscle attachments, correct vascularization, innervation and many other key developmental events. This dataset embraces late stages of mouse cranial development when the definitive tissue integration events take place. Without doubts, such tomographic data will be suitable for improving our understanding of these fundamental questions.

Data Availability

The dataset presented in this work is available through the *GigaScience* Database repository [17]. We provide already-reconstructed X-ray computed tomography data. The dataset is presented as 8-bit TIFF stacks of corresponding CT slices and manually segmented masks. The folders are structured so that each folder representing one sample contains two folders: Images and Masks. The Images folder contains reconstructed tomographic slices in TIFF format, the folder Masks contains corresponding manually segmented masks. The naming convention is: Sample_name.tif for slice and mask_Sample_name.tif for segmented mask. To enable the users of the dataset to visually inspect the embryo heads in 3-D, .stl files were included together with the image stacks. Additionally, a text file is provided for each sample containing information about the voxel size.

As tiff stacks, the deposited data can be opened and viewed in any basic image viewer, however, to fully take advantage of the possibilities provided by the 3-dimensional nature of the images, a specialized viewer for 3-D data is recommended. Avizo (Thermo Fisher Scientific, USA) is a commercial software providing a broad range of possibilities to visualize, manipulate and analyze 3-D µCT image

data. Another commercial software option is VG Studio MAX (Volume Graphics GmbH, Germany). We recommend the Fiji ImageJ distribution [18] as a free software option to view and manipulate the provided data. As the manually segmented masks of the data are binary images composed of 0s (background) and 1s (mesenchymal condensations/cartilage), they may be displayed as black images. To visually inspect the data, it may be necessary to set the software display window to the range from 0 to 1 in some image viewers. The included .stl files of the embryo heads may be explored in many different 3-D mesh viewers, a popular free open-source software option is MeshLab [19]. Reconstructions are also available for browsing in Sketchfab https://sketchfab.com/GigaDB/collections/mouse-embryo

Declarations

List of abbreviations

µCT	micro X-ray computed tomography
CNN	convolutional neural network
GE	General Electric
PTA	phosphotungstic acid
VG	Volume Graphics

Ethics approval and consent to participate

All animal work was approved and permitted by the Local Ethical Committee on Animal Experiments (North Stockholm Animal Ethics Committee) and conducted according to The Swedish Animal Agency´s Provisions and Guidelines for Animal Experimentation recommendations.

Consent for publication

Not applicable.

Competing interests

The authors declare no competing interests.

Funding

This research was carried out under the project CEITEC 2020 (LQ1601) with financial support from the Ministry of Education, Youth and Sports of the Czech Republic under the National Sustainability Programme II and CzechNanoLab Research Infrastructure supported by MEYS CR (LM2018110).

JM was financially supported by grant CEITEC VUT-J-20-6477.

MT was financially supported by grant CEITEC VUT-J-20-6317 and by the Brno City Municipality as a Brno Ph.D. Talent Scholarship Holder.

Authors' contributions

JM	conceptualization, writing – original draft, visualization, writing – review & editing
MT	methodology, data curation, writing – review & editing
TZ	conceptualization, writing – review & editing
MK	writing – original draft, writing – review & editing
IA	writing – original draft
JK	funding acquisition, supervision, project administration

Acknowledgements

Not applicable.

References

1. Kaucka M, Zikmund T, Tesarova M, Gyllborg D, Hellander A, Jaros J, et al.. Oriented clonal cell dynamics enables accurate growth and shaping of vertebrate cartilage. *Elife*. 2017; doi: 10.7554/eLife.25902.

2. Kaucka M, Petersen J, Tesarova M, Szarowska B, Kastriti ME, Xie M, et al.. Signals from the brain and olfactory epithelium control shaping of the mammalian nasal capsule cartilage. *Elife*. 2018; doi: 10.7554/eLife.34465.

3. Zollikofer CPE, Ponce de Leon MS. Visualizing patterns of craniofacial shape variation in Homo sapiens. *Proc R Soc B Biol Sci*. 2002; doi: 10.1098/rspb.2002.1960.

4. Metscher BD. MicroCT for developmental biology: A versatile tool for high-contrast 3D imaging at histological resolutions. *Dev Dyn*. 2009; doi: 10.1002/dvdy.21857.

5. Balto K, Muller R, Carrington DC, Dobeck J, Stashenko P. Quantification of periapical bone destruction in mice by micro-computed tomography. *J Dent Res*. 2000; doi: 10.1177/00220345000790010401.

6. Sabolova V, Brinek A, Sladek V. The effect of hydrochloric acid on microstructure of porcine (Sus scrofa domesticus) cortical bone tissue. *Forensic Sci Int*. 2018; doi: 10.1016/j.forsciint.2018.08.030.

7. Dosedelova H, Stepankova K, Zikmund T, Lesot H, Kaiser J, Novotny K, et al.. Age-related changes in the tooth–bone interface area of acrodont dentition in the chameleon. *J Anat*. 2016; doi: 10.1111/joa.12490.

8. Landova Sulcova M, Zahradnicek O, Dumkova J, Dosedelova H, Krivanek J, Hampl M, et al.. Developmental mechanisms driving complex tooth shape in reptiles. *Dev Dyn*. 2020; doi: 10.1002/dvdy.138.

9. Metscher BD. Micro CT for comparative morphology: Simple staining methods allow high-contrast 3D imaging of diverse non-mineralized animal tissues. *BMC Physiol*. 2009; doi: 10.1186/1472-6793-9-11.

10. Choi JP, Foley M, Zhou Z, Wong WY, Gokoolparsadh N, Arthur JSC, et al.. Micro-CT imaging reveals Mekk3 heterozygosity prevents cerebral cavernous malformations in Ccm2-deficient mice. *PLoS One*. 2016; doi: 10.1371/journal.pone.0160833.

11. Pai VM, Kozlowski M, Donahue D, Miller E, Xiao X, Chen MY, et al.. Coronary artery wall imaging in mice using osmium tetroxide and micro-computed tomography (micro-CT). *J Anat*. 2012; doi: 10.1111/j.1469-7580.2012.01483.x.

12. Degenhardt K, Wright AC, Horng D, Padmanabhan A, Epstein JA. Rapid 3D phenotyping of cardiovascular development in mouse embryos by micro-CT with iodine staining. *Circ Cardiovasc Imaging*. 2010; doi: 10.1161/CIRCIMAGING.109.918482.

13. Choi JP, Yang X, Foley M, Wang X, Zheng X. Induction and micro-CT imaging of cerebral cavernous malformations in mouse model. *J Vis Exp*. 2017; doi: 10.3791/56476.

14. Tesarova M, Zikmund T, Kaucka M, Adameyko I, Jaros J, Palousek D, et al. Use of micro computed-tomography and 3D printing for reverse engineering of mouse embryo nasal capsule. *J Instrum*. 2016; doi:10.1088/1748-0221/11/03/C03006.

15. Theiler K. The House Mouse: Atlas of Embryonic Development. 2nd ed. Springer-Verlag; 1989.

16. Ronneberger O, Fischer P, Brox T. U-net: Convolutional networks for biomedical image segmentation. *Medical Image Computing and Computer-Assisted Intervention - MICCAI 2015*. 2015; doi:10.1007/978-3-319-24574-4_28.

17. Matula J; Tesarova M; Zikmund T; Kaucka M; Adameyko I; Kaiser J (2021): Supporting data for "X-ray microtomography-based atlas of mouse cranial development" GigaScience Database. http://dx.doi.org/10.5524/100862

18. Schindelin J, Arganda-Carreras I, Frise E, Kaynig V, Longair M, Pietzsch T, et al.. Fiji: An open-source platform for biological-image analysis. *Nat. Methods*. 2012; doi:10.1038/nmeth.2019.

19. Cignoni P, Callieri M, Corsini M, Dellepiane M, Ganovelli F, Ranzuglia G. MeshLab: An open-source mesh processing tool. *6th Eurographics Ital Chapter Conf 2008 - Proc*. 2008; 10.2312/LocalChapterEvents/ItalChap/ItalianChapConf2008/129-136.

Figure 1

Click here to access/download;Figure;fig1.tif

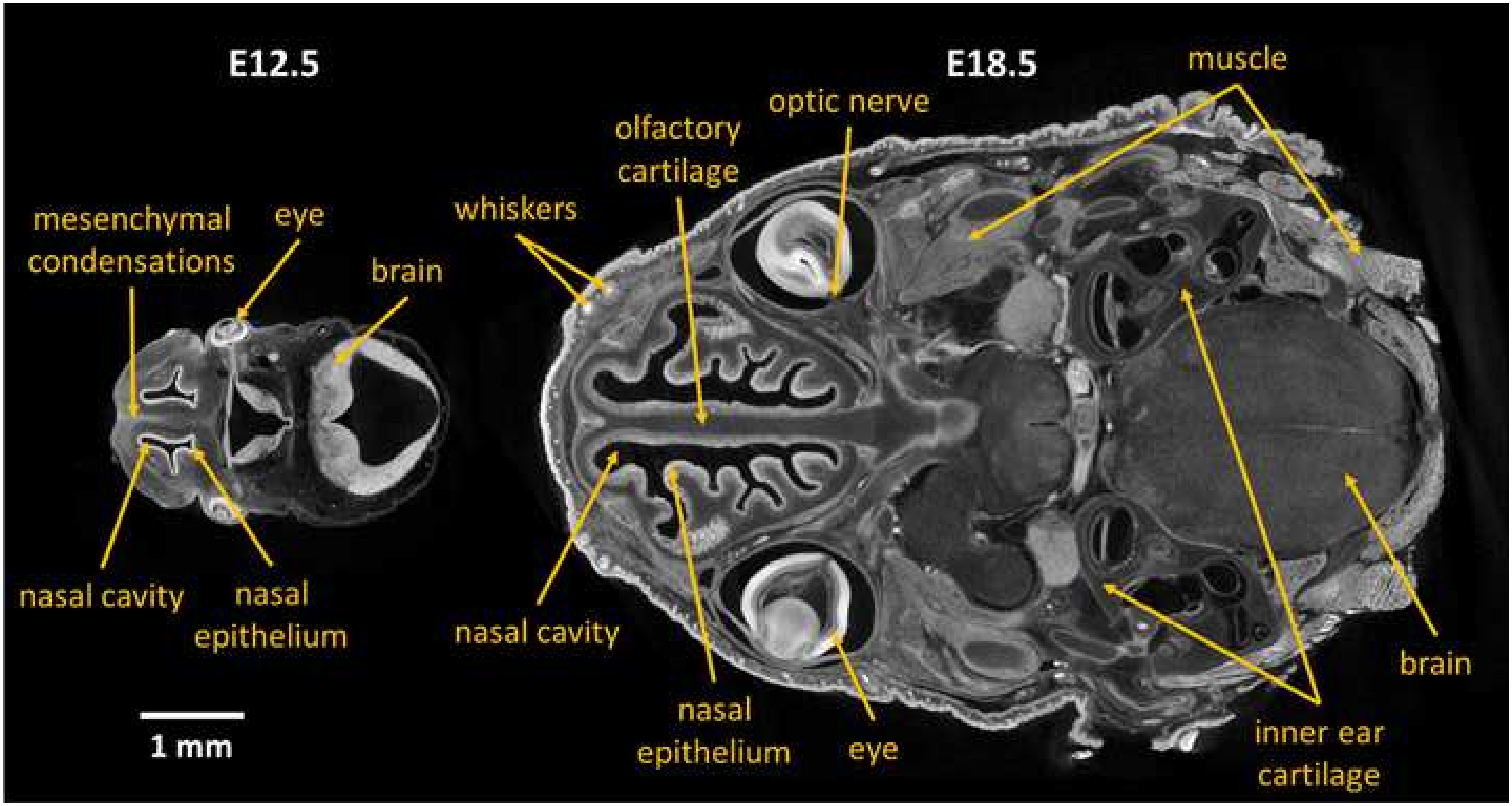

Figure 2

Click here to access/download;Figure;fig2.tif

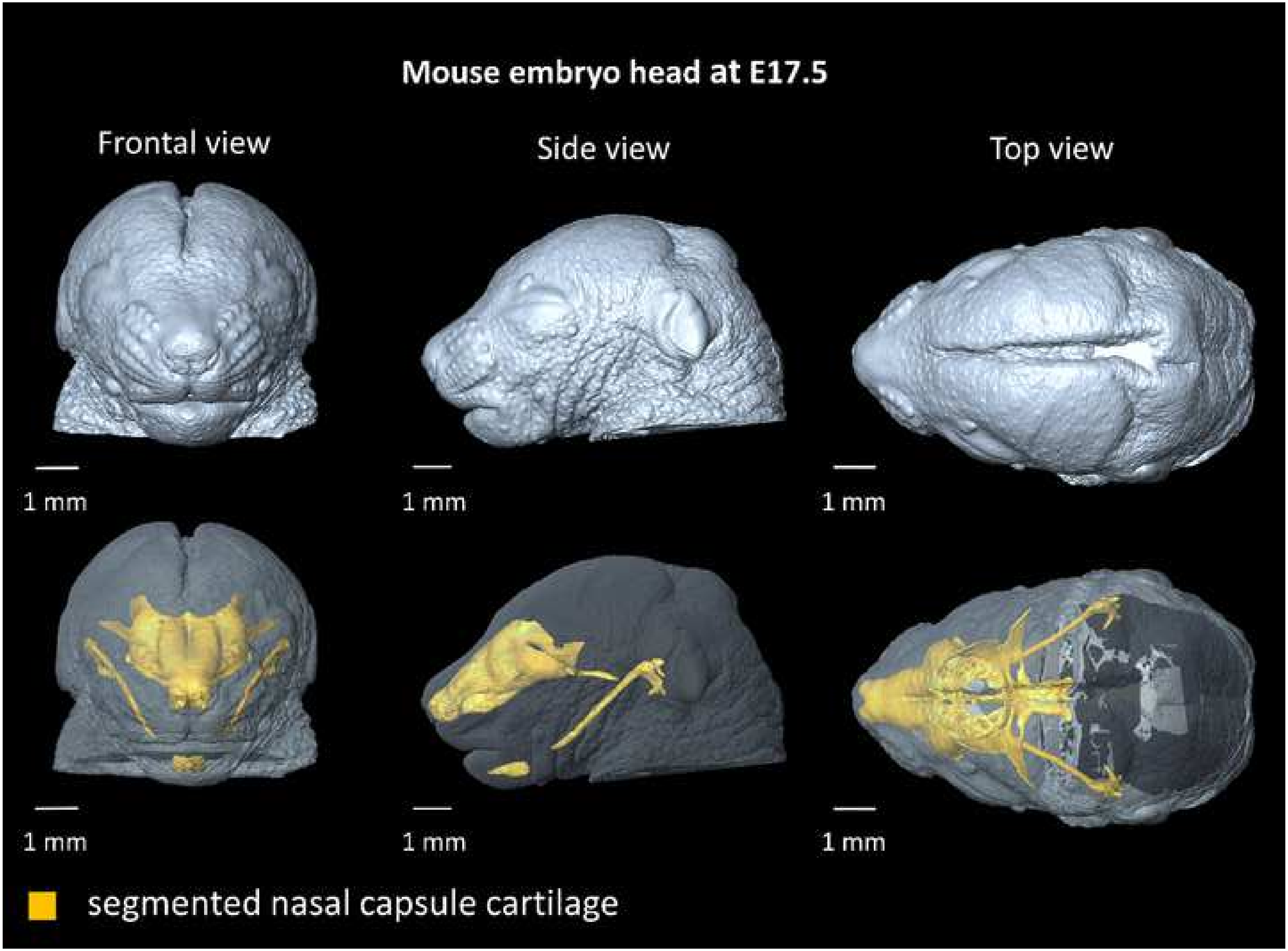

Figure 3

Click here to access/download;Figure;fig3.tif

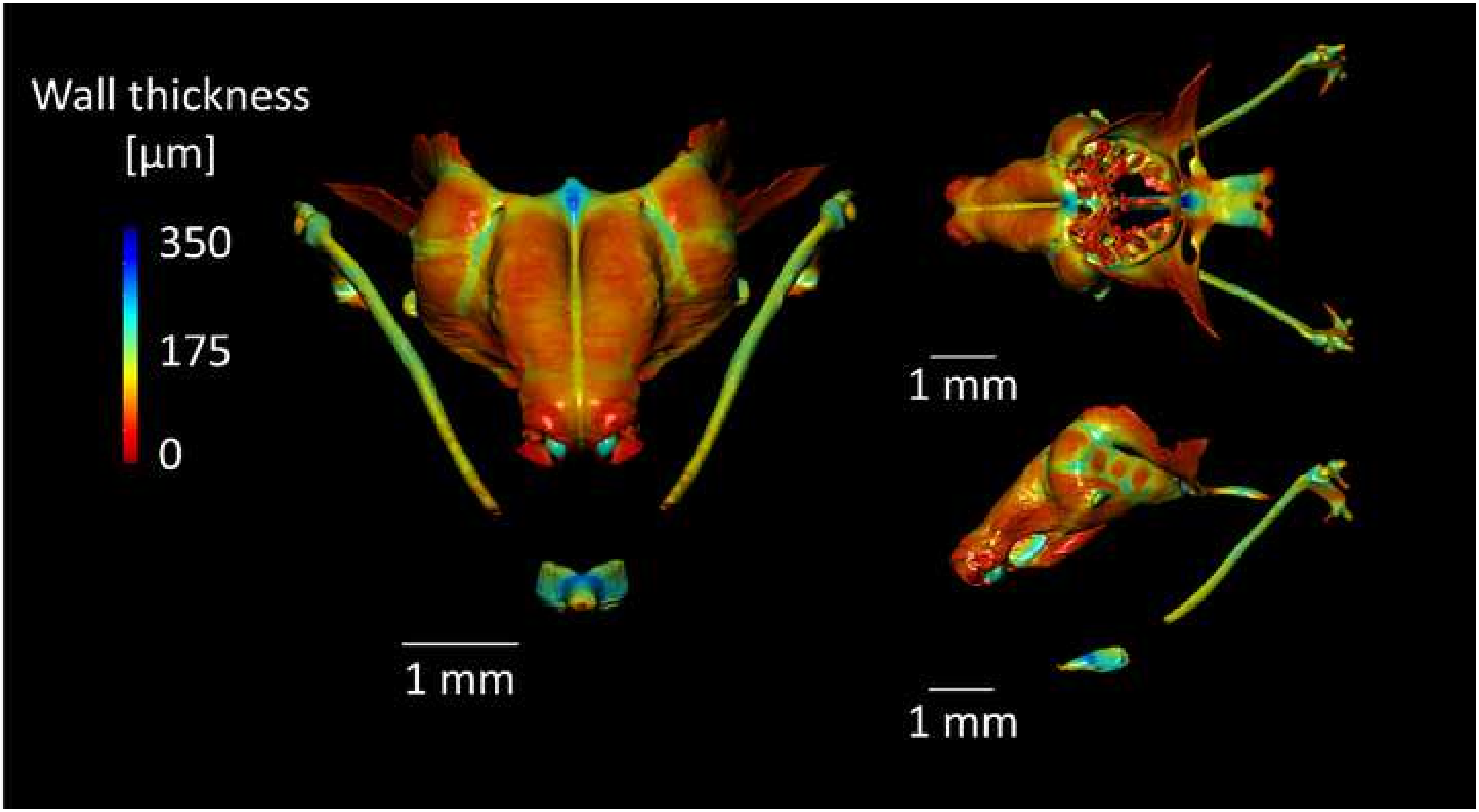

Figure 4

Click here to access/download;Figure;fig4.tif

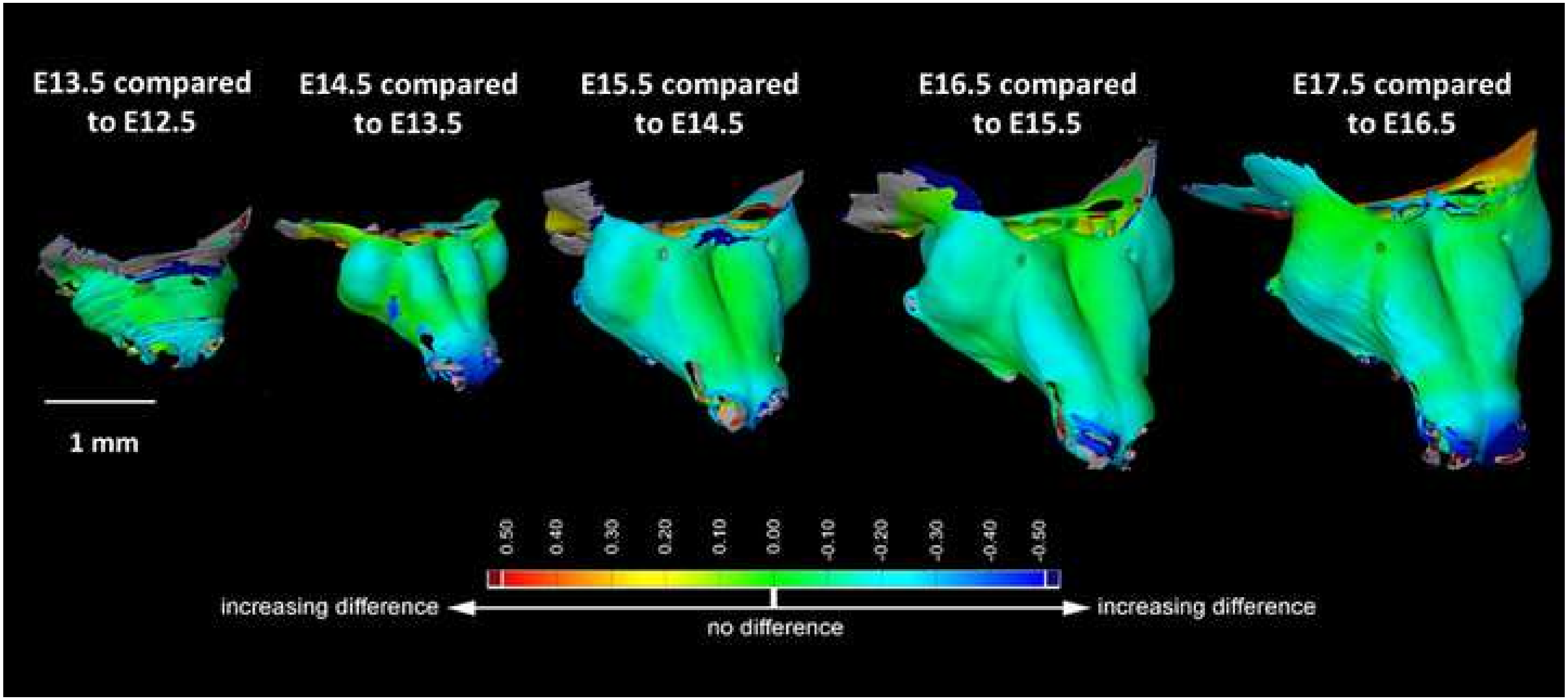

Figure 5

Click here to access/download;Figure;fig5.tif

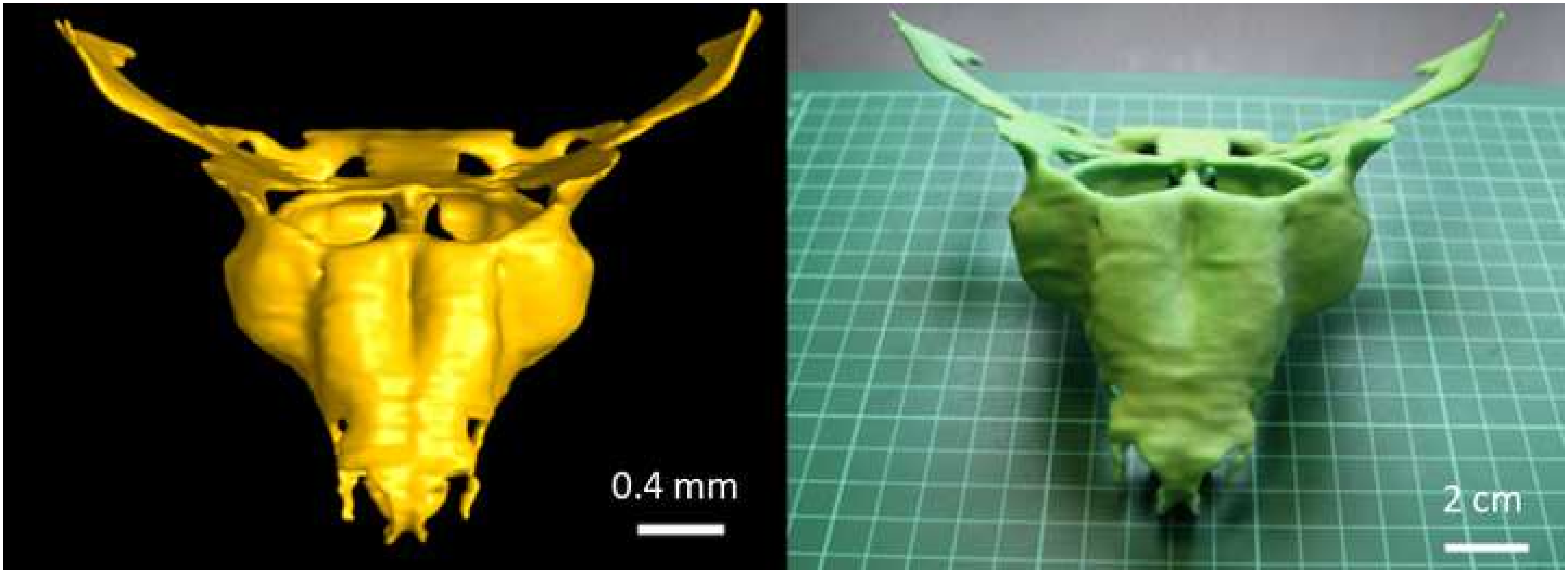

Supplement: giab012_GIGA-D-20-00282_Revision_1 [file giab012_giga-d-20-00282_revision_1.pdf]
