# Supplementary material for: X-ray microtomography–based atlas of mouse cranial development
Source: Gigascience. 2021 Mar 2;10(3):giab012. doi: 10.1093/gigascience/giab012 (PMC7936920; doi:10.1093/gigascience/giab012)
Supplement: giab012_Response_to_Reviewer_Comments_Original_Submission [file giab012_response_to_reviewer_comments_original_submission.pdf]

Author's Response To Reviewer Comments

Close

Reviewer #1: The authors present a set of 3D images of mouse embryo heads from daily stages particularly important for facial skeletal development (E12-18). These are high-quality and complete contrast-enhanced micro-CT images showing details of soft tissues at the level of low-magnification histology. They will be potentially useful in any number of embryological studies. The dataset also includes segmentations (delimited sub-volumes, or masks) of the developing facial cartilage, from another publication. This is a model submission for presenting a dataset of this kind.
In the Word document, I have offered some suggestions for slight improvements to the style and presentation. Otherwise, the report appears ready for publication.

Response: We are grateful to the reviewer for their kind words. We appreciate the suggestions in the attached word document, and we incorporated them into the manuscript where we found it appropriate.

Reviewer #2: The manuscript by Matula et al demonstrated the use of novel micro-CT imaging to visualize mouse embryo cranial development. The manuscript is well-written overall with high-quality micro-CT images. However, the authors only used one embryo in each of timepoints.
1.In sample preparation, authors mention that the embryos undergo dehydration and rehydration. These processes cause significant shrinkage and expansion of the sample. Hence, the results may present distorted structural features.

Response: We are thankful to the reviewer for this comment. The 3-D segmentation of nasal capsule and Meckel cartilage in presented in this work corresponds with visualizations of this structure by other techniques (PMID: 28414273). We are aware that shrinkage may occur, and for this reason, the dehydration and rehydration are performed as slow as possible (30%, 50% and 70% methanol for 1 day each) to minimize shrinking of the embryonic tissue. Dehydration and rehydration are a standard process when staining by PTA and it has been used for wide application in developmental studies (10.1002/dvdy.136).

Reviewer #2: Why are voxel size differ between samples? This is problematic when comparing different samples. Especially for future studies comparing morphological features between samples (i.e. different age, treatment, mouse genetic studies).

Response: The general strategy is to always obtain data of the highest possible resolution for each sample. In order to achieve the best possible resolution for each sample, the scanning parameters are modified for each sample separately because of the individual sample size. This is driven by the geometric magnification of the CT system; state-of-the-art systems provide voxel size of approximately 1/1000 of the sample size.
As almost every software designed to work with 3-D imaging data respects the given voxel size, morphological comparison between the group of scans or even scans created with completely different voxel sizes, should pose no issues. If it is necessary to have scans of the same voxel size, it is possible to resample the provided data to a unified voxel size, and still keep the resolution high, but this would degrade the quality of the image data with the highest resolution and this is not something we feel would benefit the dataset.

Reviewer #2: The study reused existing micro-CT scans to analyse mouse cranial development. The image quality is high but only one embryo was scanned in each time point. The study would be significantly improved if authors can demonstrate its variability or reproducibility by scanning multiple embryos (>3 embryos/timepoint).

Response: The goal of this published set of data is not to show variability among developmental groups, but to provide a compact, easy-to-work-with dataset, that can be quickly downloaded and processed and represents the general development of the mouse embryo in the set time range. This is further highlighted by the Theiler staging process performed per the suggestion of Reviewer #3. Furthermore, we do not try to quantify any variability in this manuscript since it is highly dependent on the specific

biological application.

Reviewer #2: Can authors show any quantification? For example, measuring the length of nasal capsule cartilage or volume of nasal cavity. The quantification is possible with micro-CT and AVIZO software

Response: We agree with the reviewer, that some general quantification of the morphology would benefit the data validation and quality control section of the manuscript. We included measurements of nasal capsule morphology in terms of its length and width (lines 154, 161-162, table 3). This also shows a general progress in the growth of the nasal capsule and introduce a potential reader to the dimensions present. More specific quantification would be again highly dependent on the particular application utilizing the data can be selected.

Reviewer #2: The study used phosphotungstic acid (PTA) as a contrast staining. Have they tested other staining methods such as Lugol's Iodine and Osmium Tetraoxide staining? These staining methods are more widely used(e.g. PMID: 27513872, 27345427, 28892037).

Response: The focus of the study utilizing this dataset is mainly the investigation of craniofacial development. PTA offers unparalleled contrast in imaging of cartilaginous tissues in comparison to the other suggested staining solutions (as the contrasting solution does not penetrate the cartilaginous tissues and as a result, visible difference can be observed in attenuation of cartilage and the surrounding tissues. The trade-off is the long staining time as it takes time for the large molecule of PTA to fully penetrate the sample. We may have neglected to mention the other, more widely utilized staining methods, which is why we added a note on these with relevant literature to the Context section of the manuscript (lines 73-74).

Reviewer #2: 1% agarose gel. Need to specify which type of agarose was used. Low temperature agarose gel should have been used to avoid any sample damage as regular agarose gel has high melting point which can destruct the sample

Response: We are grateful to the reviewer for this comment. During the revision of the manuscript, we found an error in the concentration of the utilized agarose. We deeply apologize for this mistake and corrected the value (1% agarose to 0.5% agarose). The agarose utilized in this work is A5304, Sigma-Aldrich. This agarose is not of the low-melting point type, but special care is given to make sure no damage occurs to the sample during its fixation. After its dissolution in boiling water, the agarose is left to cool to safe temperature (45 °C), while it still remains fluid and only then it is used for the sample fixation. The information about the agarose type was included to the manuscript (line 104).

Reviewer #2: There is huge difference in sample incubation in PTA solution. E12.5 was stained for 7 days, E15.5 for 21 days, E18.5 for 49 days. Can authors optimise the incubation procedure to reduce incubation time? Perhaps using different contrast staining methods (i.e. Lugol's Iodine or Osmium Tetraoxide)

Response: It is true the staining times are very high in comparison to other staining methods. The answer to the reviewer's question about the long staining time was partially answered in response to the minor issue #1. The staining used for the presented samples was focused on making the nasal capsule as distinguishable as possible for the operator to be able to perform the manual segmentation of the mesenchymal condensations/cartilage. We also experimented with the contrast staining methods the reviewer mentions, but we achieved the greatest contrast with PTA (phosphotungstic acid) with the obvious trade-off of much longer staining times, to allow the contrast to fully penetrate the tissues due to its large molecule size.

Reviewer #2: How were the micro-CT scanning parameters determined for optimal quality? Also, how long was each scan?

Response: The scanning time with the 2000 projections and 900 ms exposure time with triple averaging of the X-ray projections was about 1 h 30 min. The information about the scanning time was included to the manuscript (lines 120-121). These scanning parameters are a result of optimization to achieve the best possible image quality with a reasonable scanning time. We experimented with several setups of X-ray tube (accelerating voltage, current and filtration) and detector (averaging, number of projections, exposure time). The resulting data quality were evaluated terms of contrast, which allowed precise segmentation, and the signal-to-noise ratio.

Reviewer #2: Line 130-132 “This three-fold increase in segmentation speed does not affect the accuracy of the segmentation in a significant way...”. Needs referencing or data to support the statement.

Response: To support this statement, we performed an experiment with a manually segmented cartilaginous nasal capsule, that was segmented on a slice-by-slice basis. From this data, we selected only every 3rd slice in the plane, where the segmentation was performed and then utilized the same interpolation process as was used in this work. The result of this experiment is that there is a 98% overlap (Dice coefficient) between the volume utilizing interslice interpolation and dataset utilizing slice-by-slice segmentation in the case of this type of sample. This information was added to the manuscript (lines 138-140).

Reviewer #3: This Data Note describes a microCT dataset of mouse embryonic development. The image data is of high quality and high contrast, and consists of 8-bit TIFF stacks with an impressive 3-micron isotropic voxel resolution for the E13.5 specimen. The authors are to be commended for using the contrast agent phosphotungstic acid to improve the quality of the microCT images. In addition, the authors provide manually segmented masks of mesenchymal condensations and nasal capsule cartilage, and the authors claim that these can be used to measure and understand morphological features of cranial development, such as chondrocranium fusion events. The authors further highlight the reuse potential of the manually segmented masks for developing machine-learning approaches for automated segmentation. I think that this is an interesting use case for these data, and I commend the authors for making these data publicly available in the GigaScience DataBase.
Major comment 1 The authors highlight the reuse potential of the microCT data for exploring developmental changes in craniofacial morphology between E12.5 and E18.5. On this note, there are existing histological atlases detailing craniofacial development, such as Kaufman's 'The Atlas of Mouse Development', that use Theiler staging, which is a morphological staging system developed by Karl Theiler to accurately stage mouse embryos. The reason why a morphological staging system is important is because in a litter of mouse embryos of the same age, some embryos are observably more advanced in development than others. One method of handling this inherent biological variation that one observes in age-matched littermates is to further stage the embryos based on morphological criteria. With this in mind, I invite the authors to Theiler stage each of the seven mouse embryo models in this dataset. This would be a great asset and would allow a researcher to compare the microCT data outlined in this Data Note to stage-matched anatomical atlas models, such as those used in the Kaufman Atlas and the eHistology resource (https://www.emouseatlas.org/emap/eHistology/). Of note, online resources are available to assist in the Theiler staging process: https://www.emouseatlas.org/emap/ema/theiler_stages/StageDefinition/stagedefinition.html In addition, Karl Theiler's book on Theiler staging is openly available at the following link: https://www.emouseatlas.org/emap/ema/theiler_stages/house_mouse/book.html

Response: We would like to thank the reviewer for this very helpful suggestion and for providing the links to relevant resources. We see how important this type of staging is for further re-use of this data. For this reason, we performed Theiler staging and incorporated the information about the embryos Theiler stages into the manuscript (Table 1, column Theiler stage, lines 107-110).

Reviewer #3: Whereas I can open the TIFF stacks of the microCT image data in Fiji / ImageJ and I can observe grayscale image data, the corresponding mask image data just appears as a black image. Can the authors provide instructions on how to view the manually segmented masks using Fiji / ImageJ? The example dataset I am using for Fiji / ImageJ is the E13.5 dataset.

Response: As the masks are saved as 8-bit tiff image and contains only values 0 (background) and 1 (mesenchymal condensation/cartilage), it will be, by many image viewers, displayed as almost black image, because they are using the whole range from 0 to 255 to display the image. To view the segmentation, it is necessary to set the display window in the selected software to the range 0 to 1. The segmentation will then be displayed as white on black background. We see how this could be confusing for potential users of the data. We modified the data re-use section of the manuscript with this information (219-222).

Reviewer #3: Figure 2 shows a 3D surface reconstruction of an E17.5 mouse embryo head. I see great value in surface reconstructions of the 3D models as they allow researchers to explore the external morphology of each of the specimens. Can the authors please clarify whether they will be submitting 3D surface reconstructions (e.g. STL format) of all seven models to the GigaScience DataBase?

Response: We agree with the reviewer, that seeing the 3-D rendering of the embryos will help potential

re-users of this dataset. We therefore include 3-D .stl files alongside the data showing the embryo heads. Appropriate text was added to the data availability section of the manuscript on how to work with these files (lines 210-211, 222-223).

In addition to the reviewers comments, two errors in the manuscript were found during the revision process. Firstly, in the rehydratation part of the staining protocol, ethanol was mistakenly stated instead of methanol. This error in the manuscript was corrected on line 103. Additionally, when stating voxel size of the Embryo 1 sample, by error we used a value of 3.2 µm. We corrected this value to the correct value of 2.6 µm in the Table 2 of the manuscript. This error was also corrected in the submitted data. We deeply apologize for these mistakes. Furthermore, we would like to express our gratitude to the reviewers for their helpful comments.

Close
