# Supplementary material for: X-ray microtomography–based atlas of mouse cranial development
Source: Gigascience. 2021 Mar 2;10(3):giab012. doi: 10.1093/gigascience/giab012 (PMC7936920; doi:10.1093/gigascience/giab012)
Supplement: giab012_Reviewer_3_Report_Original_Submission — Chris Armit -- 11/3/2020 Reviewed [file giab012_reviewer_3_report_original_submission.pdf]

Reviewer Report

Title: X-ray microtomography-based atlas of mouse cranial development

Version: Original Submission **Date:** 11/3/2020

Reviewer name: Chris Armit

Reviewer Comments to Author:

This Data Note describes a microCT dataset of mouse embryonic development. The image data is of high quality and high contrast, and consists of 8-bit TIFF stacks with an impressive 3-micron isotropic voxel resolution for the E13.5 specimen. The authors are to be commended for using the contrast agent phosphotungstic acid to improve the quality of the microCT images. In addition, the authors provide manually segmented masks of mesenchymal condensations and nasal capsule cartilage, and the authors claim that these can be used to measure and understand morphological features of cranial development, such as chondrocranium fusion events. The authors further highlight the reuse potential of the manually segmented masks for developing machine-learning approaches for automated segmentation. I think that this is an interesting use case for these data, and I commend the authors for making these data publicly available in the GigaScience DataBase.

Major comment 1

The authors highlight the reuse potential of the microCT data for exploring developmental changes in craniofacial morphology between E12.5 and E18.5. On this note, there are existing histological atlases detailing craniofacial development, such as Kaufman's 'The Atlas of Mouse Development', that use Theiler staging, which is a morphological staging system developed by Karl Theiler to accurately stage mouse embryos. The reason why a morphological staging system is important is because in a litter of mouse embryos of the same age, some embryos are observably more advanced in development than others. One method of handling this inherent biological variation that one observes in age-matched littermates is to further stage the embryos based on morphological criteria.

With this in mind, I invite the authors to Theiler stage each of the seven mouse embryo models in this dataset. This would be a great asset and would allow a researcher to compare the microCT data outlined in this Data Note to stage-matched anatomical atlas models, such as those used in the Kaufman Atlas and the eHistology resource (https://www.emouseatlas.org/emap/eHistology/).

Of note, online resources are available to assist in the Theiler staging process:

https://www.emouseatlas.org/emap/ema/theiler_stages/StageDefinition/stagedefinition.html

In addition, Karl Theiler's book on Theiler staging is openly available at the following link:

https://www.emouseatlas.org/emap/ema/theiler_stages/house_mouse/book.html

Major comment 2

Whereas I can open the TIFF stacks of the microCT image data in Fiji / ImageJ and I can observe grayscale image data, the corresponding mask image data just appears as a black image. Can the authors provide instructions on how to view the manually segmented masks using Fiji / ImageJ? The example dataset I am using for Fiji / ImageJ is the E13.5 dataset.

Major comment 3

Figure 2 shows a 3D surface reconstruction of an E17.5 mouse embryo head. I see great value in surface reconstructions of the 3D models as they allow researchers to explore the external morphology of each of the specimens. Can the authors please clarify whether they will be submitting 3D surface reconstructions (e.g. STL format) of all seven models to the GigaScience DataBase?

Level of Interest

Please indicate how interesting you found the manuscript: Choose an item.

Quality of Written English

Please indicate the quality of language in the manuscript: Choose an item.

Declaration of Competing Interests

Please complete a declaration of competing interests, considering the following questions:

- Have you in the past five years received reimbursements, fees, funding, or salary from an organisation that may in any way gain or lose financially from the publication of this manuscript, either now or in the future?
- Do you hold any stocks or shares in an organisation that may in any way gain or lose financially from the publication of this manuscript, either now or in the future?
- Do you hold or are you currently applying for any patents relating to the content of the manuscript?
- Have you received reimbursements, fees, funding, or salary from an organization that holds or has applied for patents relating to the content of the manuscript?
- Do you have any other financial competing interests?
- Do you have any non-financial competing interests in relation to this paper?

If you can answer no to all of the above, write 'I declare that I have no competing interests' below. If your reply is yes to any, please give details below.

I declare that I have no competing interests.

I agree to the open peer review policy of the journal. I understand that my name will be included on my report to the authors and, if the manuscript is accepted for publication, my named report including any attachments I upload will be posted on the website along with the authors' responses. I agree for my report to be made available under an Open Access Creative Commons CC-BY license (http://creativecommons.org/licenses/by/4.0/). I understand that any comments which I do not wish to be included in my named report can be included as confidential comments to the editors, which will not be published.

Choose an item.

To further support our reviewers, we have joined with Publons, where you can gain additional credit to further highlight your hard work (see: https://publons.com/journal/530/gigascience). On publication of this paper, your review will be automatically added to Publons, you can then choose whether or not to claim your Publons credit. I understand this statement.

Yes Choose an item.
